# A Guide to Nucleic Acid Vaccines in the Prevention and Treatment of Infectious Diseases and Cancers: From Basic Principles to Current Applications

**DOI:** 10.3389/fcell.2021.633776

**Published:** 2021-05-25

**Authors:** Furong Qin, Fan Xia, Hongli Chen, Bomiao Cui, Yun Feng, Ping Zhang, Jiao Chen, Min Luo

**Affiliations:** ^1^State Key Laboratory of Biotherapy and Cancer Center, West China Hospital, Sichuan University, Chengdu, China; ^2^Department of Neurosurgery, West China Hospital, Sichuan University, Chengdu, China

**Keywords:** DNA vaccines, RNA vaccines, infectious diseases, cancer, nucleic acid vaccines

## Abstract

Faced with the challenges posed by infectious diseases and cancer, nucleic acid vaccines present excellent prospects in clinical applications. Compared with traditional vaccines, nucleic acid vaccines have the characteristics of high efficiency and low cost. Therefore, nucleic acid vaccines have potential advantages in disease prevention and treatment. However, the low immunogenicity and instability of nucleic acid vaccines have limited their development. Therefore, a large number of studies have been conducted to improve their immunogenicity and stability by improving delivery methods, thereby supporting progress and development for clinical applications. This article mainly reviews the advantages, disadvantages, mechanisms, delivery methods, and clinical applications of nucleic acid vaccines.

## Introduction

Since the first vaccines were developed over 200 years ago, vaccines have directly decreased the morbidity and mortality caused by dangerous diseases across large human populations (Rappuoli et al., 2011; Koff et al., 2013). Furthermore, vaccines can be prophylactic or therapeutic in clinical practice and can be broadly divided into live attenuated vaccines (weakened microorganisms), inactivated vaccines (killed microorganisms), toxoid vaccines (inactivated bacterial toxins), and subunit vaccines (purified antigens) (Wadhwa et al., 2020). To date, conventional vaccines have had a crucial impact on reducing the burden of numerous infectious diseases. For instance, they eradicated mallpox and evidently restricted the incidence of diseases, including tetanus, polio, diphtheria, and measles worldwide (Younger et al., 2016). Despite these achievements, there remain limitations and potential associated problems for the conventional methods. The risk that attenuated antigens may revert to full virulence is quite low, rendering this method unfavorable for highly pathogenic viruses. In addition, only under precisely controlled and characterized conditions can live attenuated vaccines induce the required protective immunity to prevent obvious disease symptoms in the host animal. Inactivated vaccines have certain restrictions related to the method of presentation, resulting in a limited immune response, which requires adjuvants or immunostimulants to enhance the response. Additionally, live attenuated vaccines and inactivated vaccines may face the challenge of production, since the requirement for a high biosafety level and dedicated laboratories for cultivation. It can effectively avoid the inclusion of undesired “foreign” protein from the culture medium, such as eggs, tissue culture, or simply culture medium, which may have an impact on immunogenicity or be potentially allergenic/reactogenic. On account of the inadequate immunogenicity of the protein antigen alone, subunit vaccines and recombinant protein-based vaccines are typically used in combination with adjuvants or delivery systems to elicit a protective effect. Moreover, the constant emergence of novel pathogens, as well as the re-emergence of known pathogens, requires investigators to develop new vaccines in a manner that allows for the rapid development of safe and effective vaccines. Therefore, scientists have found that nucleic acid vaccines are becoming a robust and versatile technical strategy for combatting infectious diseases and cancers.

Nucleic acid vaccines have the potential to be safe, effective, and cost-effective. Moreover, the immune responses induced by nucleic acid vaccines only target the selected antigen in the pathogen. Nucleic acid-based vaccines, including DNA (as plasmids) and RNA [as messenger RNA (mRNA)] vaccines, exhibit promising potential in targeting various indications and diseases. Furthermore, cancer vaccines present an attractive strategy that can induce specific and persistent immune responses against tumor antigens. DNA vaccines are based on bacterial plasmids that encode antigens and immunostimulatory molecules (i.e., IL-2 and GM-CSF). The first case of DNA vaccine-mediated immunity began in the 1990s, when plasmid DNA encoding the influenza A nucleoprotein led to a protective and specific cytotoxic T lymphocyte (CTL) response (Yankauckas et al., 1993). Additionally, several animal models have successfully demonstrated that DNA vaccines can be used to prevent or treat allergies, autoimmunity, infectious diseases, and cancer (Wolff et al., 1990; Ulmer et al., 1993; Fuller and Haynes, 1994; Donnelly et al., 1996; Wang et al., 2008). Likewise, early successful use of *in vitro* transcribed (IVT) mRNA in animals began in 1990. At that time, the gene of encoding the mRNA sequence was injected into mice, and then researchers detected the produced protein (Wolff et al., 1990).

In this review, we first provide an overview of current understanding of the nucleic acid vaccines. We next focused on the delivery of nucleic acid vaccines, with an emphasis on improving efficacy. Finally, we turned our attention to the clinical applications of nucleic acid vaccines by elaborating on the prospects and needed improvements.

## DNA Vaccines

DNA vaccines are generated by inserting a gene encoding antigens into a bacteria-derived plasmid, which needs to be controlled by a powerful promoter [in most cases, the CMV- promoter (Leitner et al., 2001)]. DNA plasmids are replicated in bacteria, which can be selected based on antibiotic resistance mediated by genes carrying resistance markers, using the prokaryotic origin of replication. Additionally, DNA vaccines can affect not only humoural immunity but also cellular immunity. Although the precise mechanisms underlying the induction of an immune response to antigens expressed by host cells following DNA immunization have not yet been determined, we have a considerable understanding of the roles of immune cells in the processing, presentation, and recognition of antigens.

### Mechanism of Action

DNA vaccines can induce both humoural and cellular immune responses, and DNA can be delivered through a variety of routes, including intramuscular (IM), intradermal (ID), mucosal, and transdermal delivery. For DNA vaccines, after internalization, the DNA is transferred to the nucleus for transcription and then translated in the cytoplasm (Bai et al., 2017).

**FIGURE 1:**
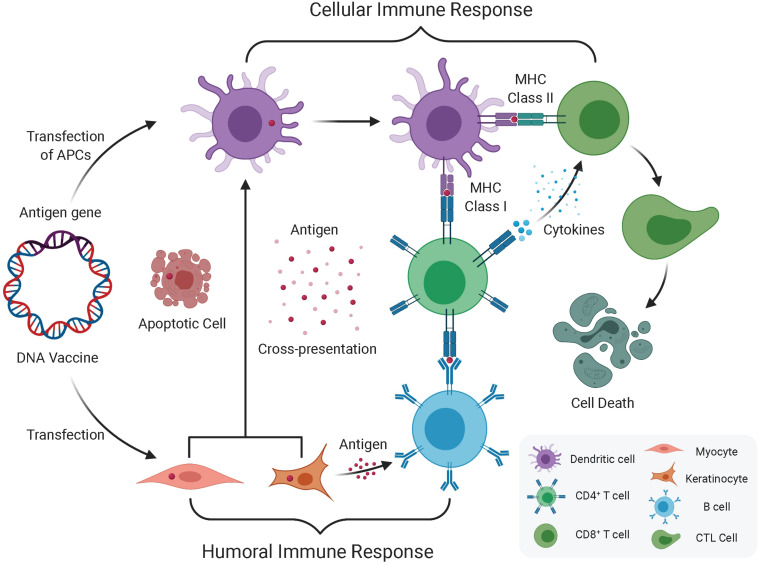
Humoral and cellular immune response induced by DNA vaccination. The immune response induced by DNA vaccine encoded antigen mainly via direct transfection of APC (DC) and Keratinocyte or Myocyte. One route that DNA vaccines transfected into keratinocytes or myocytes expresses antigen genes through exosomes or apoptotic bodies and releases derived peptides and proteins, which are then endocytosed by dendritic cells (DC), and then preferentially presents antigens to CD4^+^ T cells through MHC II, which produces cellular immune system. The other route that direct transfection of APC leads to endogenous antigen gene expression, which in turn is expressed in parallel by MHC I and MHC II, and simultaneously elicits CD8^+^ and CD4^+^ T cell responses. In addition to this cellular immune response, once the B cell receptor recognizes protein antigens from somatic cells (i.e., keratinocytes or myocytes) and obtains the help of pre-activated antigen-specific CD4^+^ T cells, which can induce a humoral immune response.

Regarding antigen presentation, the following three possible mechanisms are proposed (Figure 1): (1) after internalization, plasmid DNA is expressed by somatic cells (e.g., myocytes) and presented to CD8^+^ T cells through major histocompatibility complex (MHC) class I complexes on the somatic cells; (2) antigen presentation relies on professional antigen-presenting cells (APCs), for instance, dendritic cells (DCs), that are transfected by the plasmid DNA at the injection site, and then present the expressed antigens to T cells through MHC class I and II complexes; and (3) professional APCs phagocytose plasmid-transfected somatic cells, resulting in cross-priming and presentation of antigens to both CD4^+^ and CD8^+^ T cells. At muscle sites, since the presentation of antigens through MHC class II is needed to induce CD4^+^ helper T cells, cross-presentation is probably the major route, possibly through processing of apoptotic cell debris (Albert et al., 1998). Even in skin sites, which allow direct presentation of antigens, a gene gun can deliver DNA directly into Langerhans cells, and cross-presentation from keratinocytes is probably the main route (Stoitzner et al., 2006).

### Advantages and Disadvantages of DNA Vaccines

First, the construction of DNA vaccines is quite simple. For instance, synthetic and PCR methods result in simple engineering design modifications, and DNA vaccine plasmids are non-living, non-replicating, and non-transmitting, which can mitigate the risk of transformation into a pathogenic form or secondary infection to some extent. Therefore, without the influence of neutralizing antibody reactions, the simplicity of their composition can confer immunological advantages. Second, DNA vaccines have a primary advantage in their ability to induce local expression of target antigens and subsequently trigger antigen-specific T and B cell responses (Kutzler and Weiner, 2008), which occur because the encoded antigen can be presented by MHC Class I and Class II, thereby activating CD4^+^ and CD8^+^ T cells and indirectly activating humoural immunity. Thus, DNA vaccines not only retain many encoded safety features but also retain the specificity of subunit vaccines. Third, safety is another advantage of DNA vaccines. Prior research has clearly demonstrated the safety of DNA vaccines in animal models and human clinical trials (Trimble et al., 2009; Yuan et al., 2009; Staff et al., 2011). Unlike live attenuated vaccines, DNA vaccines have not produced obvious adverse reactions or toxicity in clinical trials, and DNA vaccines do not elicit anti-DNA antibodies, which makes repeated administration a feasible method (Yang et al., 2014). Fourth, DNA vaccines are easy to store and are more resistant to high temperatures than conventional vaccines. Moreover, DNA vaccines can be manufactured on a large scale. In addition to the above advantages, DNA vaccines can co-deliver antigen genes and a certain number of genes that modify the immune response (Prud’homme, 2005), such as cytokine genes (i.e., GM-CSF, TNF, and FLT3L) or non-cytokine genes (i.e., CD40L, IgG-Fc, and CD152). As features mentioned above, the simplicity, safety, effectiveness, and low- cost of DNA vaccines have made this vaccine approach an appealing option for current researchers.

Furthermore, there is a potential advantage related to the CpG component of plasmid vectors used in DNA vaccines for solving the issue of tumor resistance to immunity, which rapidly develops once tumors start growing (Pardoll, 2003; Prud’homme, 2004). TGF-β and IL-10, which are produced by tumors, induce the differentiation of regulatory T (Treg) cells (Groux, 2003; Horwitz et al., 2003; Sanjabi et al., 2017), or suppress immunity in other ways. Treg cells from CD4^+^ CD25^–^ T cells are induced and increase in number in cancer patients (Torgerson and Ochs, 2002), and in experimental tumor models, the application of monoclonal antibodies (mAbs) to deplete these cells is associated with increased immunity against tumor antigens and improved survival rates (Morse et al., 2002; Nishikawa and Sakaguchi, 2010). It has been reported that the treatment of antigen-loaded DCs with CpG may destroy CD8^+^ cell tolerance, while DC immunization without CpG treatment can break tolerance only after removal of Treg cells (Yang et al., 2004), which indicates that the CpG component of plasmid vectors may diminish the effects of Treg cells. Currently, considerable reports turn attentions on how CpG motifs limit the immunosuppressive function of Treg cells. It is known that CpG motifs can be recognized by Toll-like receptor-9 (TLR9), which elicits the innate immune response via activation (Hemmi et al., 2000; Medzhitov, 2007). Recent studies showed that CpG motifs as immunostimulatory agents can stimulate human plasmacytoid dendritic cells (PDCs), accelerating their maturation and survival (Hartmann et al., 1999; Bauer et al., 2001; Kadowaki et al., 2001). Moseman et al. (2004) found that CpG oligodeoxynucleotides (CpG ODNs) promote human PDC-mediated differentiation of Treg cells from naive CD4^+^ CD25^–^ T cells to CD4^+^ CD25^+^ Treg cells, which potently limit autologous and allogeneic T cell proliferation (Moseman et al., 2004). On this basis, CpG motifs can activate the TLR9 expressed on the PDC and promote differentiation of Treg cells through direct contact between PDC and T cells. Thus, the CpG content provides promising prospects for Treg cells depletion or neutralization to improve vaccination efficacy.

However, there still exist some barriers to DNA vaccines applications. One limitation of DNA vaccines is relatively low immunogenicity profiles, which impede the desired clinical application. A technical challenge associated with DNA vaccines is ensuring delivery into the cell nucleus, where antigen transcription occurs before nuclear export and translation into protein in the cytoplasm. In addition, controversial points relevant to the safety of DNA vaccines are worth for more attentions. For instance, the risk of autoimmune diseases may happen due to anti-DNA antibody. Previous studies showed that hepatitis B virus (HBV) vaccine induced anti-DNA antibody, and triggered autoimmune syndromes while combination with required adjuvants (Lilic and Ghosh, 1994; Zafrir et al., 2012). In contrast, other reports suggested that no direct evidence of DNA vaccines triggering autoimmune diseases (Mor et al., 1997). Moreover, DNA vaccines have the potential risk of integration into the host genome, which may lead to insertional mutations. Such mutations can cause a gene to dysfunction or inactivate (i.e., a tumor suppressor gene) (Würtele et al., 2003). It is reported that minicircle DNA and ministring DNA had less risk of insertional mutagenesis due to the removal of the major bacterial DNA (i.e., the unmethylated CpG repeats functioning as PAMPs), which suggested that such non-viral gene delivery vectors not only provided enhanced transgene expressions, but also were safer with less risk of insertional mutagenesis (Chen et al., 2003; Kay et al., 2010; Sum et al., 2015; Colluru et al., 2016; Munye et al., 2016; Wong et al., 2016; Monjezi et al., 2017).

## RNA Vaccines

**FIGURE 2:**
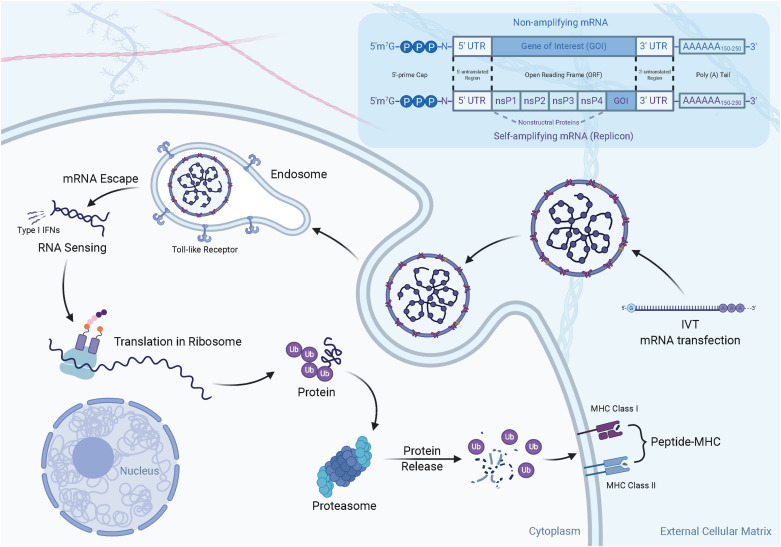
The structure and mechanism of action of mRNA vaccines. (1) There are two widely acknowledged types of mRNA vaccines, namely non-amplifying mRNA and self-amplifying mRNA. The typical components of the two mRNAs are: the cap, untranslated regions (UTRs) of 5′ and 3′, an open reading frame (ORF, including GOI) encoding the antigen, and Poly(A)n tail. Compared to non-amplifying mRNA, the size of self-amplifying mRNAs is fairly larger than non-amplifying mRNAs. For example, in α-virus-based replicons, the extra length comes from a large ORF encoding four non-structural proteins (nsP1-4). (2) *In vivo* transcribed mRNA (IVT mRNA) is obtained from a DNA template in a cell-free system. The IVT mRNA is transfected into dendritic cells (DC) by endocytosis. The mRNA is translated into antigenic proteins by utilizing a ribosome translation mechanism. The post-translational antigen protein undergoes post-translational modification and can play a role in the cells it produces. The antigen protein is degraded by the proteasome in the cytoplasm. The resulting antigen peptide is loaded onto the MHC molecule. The loaded peptide-MHC epitope complex appears on the cell surface, and after T cell receptor recognition and appropriate co-stimulation, an antigen-specific CD8^+^ T cell response is eventually induced.

Currently, there are two widely acknowledged forms of mRNA vaccines, namely, non-amplifying mRNA and self-amplifying mRNA, which are classified due to the difference in mechanisms. In terms of structure (Figure 2), non-amplifying mRNA vaccines mainly contain five critical elements for the life cycle and expression: the “cap” [m^7^Gp_3_N(N: any nucleotide)], which is a 7-methyl-guanosine residue (m7G) bound to the 5′-end of the RNA transcript via a 5′–5′ triphosphate bond with any nucleotide; a 5′ untranslated region (5′UTR) that sits immediately upstream of the translation initiation codon; an open reading frame (ORF) encoding the gene of interest (GOI); a 3′ untranslated region (3′UTR); and a tail of 100–250 adenosine residues [poly(A) tail] (Banerjee, 1980; Wickens, 1990; Dominski and Marzluff, 1999) (Figure 2). Among these elements, the cap structure is essential for stabilizing mRNA against exonucleolytic decay and making translation efficient (Gallie, 1991; Parker and Song, 2004; Yamashita et al., 2005). Similarly, the details of the poly(A) tail also affect mRNA stabilization and translation (Munroe and Jacobson, 1990; Gallie, 1991), while the untranslated regions (UTRs) are recognized by the translational machinery (ribosome) (Xiong et al., 2018). In comparison with replication-deficient mRNA constructs, self-amplifying mRNA was developed to extend the duration and amplitude of GOI expression. In addition to the five essential elements mentioned above, a self-amplifying RNA not only encodes antigens but also has a sequence similar to that of a replication-competent virus, allowing it to replicate in cells and increase protein expression. For example, a self-amplifying RNA derived from an α-virus genome contains non-structural genes (nsP1-4), a subgenomic promoter, and a variable GOI that replaces the coding sequence for the viral structural proteins (Figure 2).

### Mechanism of Action

RNA vaccines can effectively carry antigen-encoding mRNA to APCs directly *in vivo*. When antigen-encoding mRNAs are delivered into APCs in efficient ways (i.e., via nanocarriers), the mRNAs can be released and translated into relative antigenic proteins in the cytoplasm. Then, they are processed into peptide epitopes, which are subsequently combined with the MHC class I via a cross-presentation pathway. In this part, the activation of CD8^+^ T cells results from the transfer of MHC-peptides complexes to the cell surface of APCs, which leads to a corresponding immune response (Figure 2).

The pharmacodynamic activity of both native mRNA and IVT mRNA occurs in the cytoplasm. However, native mRNA is transcribed from DNA in the nucleus and crosses the nuclear membrane into the cytoplasm, while IVT mRNA enters the cytoplasm from extracellular sources (Miliotou and Papadopoulou, 2020). Once delivered into the cytoplasm, IVT mRNA will follow the same mechanisms that regulate the stability and translation of endogenous mRNA (Wadhwa et al., 2020). Therefore, mature protein products, which contain antigens, are likely to cause pathogen-specific humoural and cellular immune responses (Maruggi et al., 2019).

### Advantages and Disadvantages of RNA Vaccines

RNA vaccines, the simplest nucleic acid vaccines, are a promising alternative platform for vaccine development (Leitner et al., 2001). As for advantages, first, RNA vaccines only encode the gene of interest, which shows simplicity. Second, because mRNA can be easily and inexpensively mass-produced *in vitro*, the low price makes RNA vaccines an appealing method of treatment. Third, RNA vaccines have no risks of genome integration due to degradations *in vivo*, which may activate latent oncogenes (Karikó et al., 2008). In addition, there is no need to design expression vectors for delivery (Tan and Sun, 2018). Fourth, there is a potential safety benefit for mRNA vaccines, which have relatively low inherent immunogenicity. Nucleoside modifications not only reduce innate immune activation but also increase the translation and stability, which indicate that mRNA vaccination with modified nucleosides is a promising approach (Karikó et al., 2008; Karikó et al., 2011; Thess et al., 2015). Finally, instead of transcription in the nucleus, RNA vaccines undergo mRNA translation into protein in the host cell cytoplasm after direct injection, thereby avoiding nuclear membrane barriers.

In fact, due to the instability of RNA vaccines, the expression of antigens is transient after RNA delivery. The three major challenges in the delivery of RNA vaccines are (1) RNase-mediated degradation, (2) high molecular weight, and (3) electrostatic repulsion resulting from the interaction between the negatively-charged mRNA molecules and the negative charge of the proteoglycan-coated cell membrane (Schlake et al., 2019), which leads to failed passive diffusion across the plasma membrane.

## Progress in Nucleic Acid Vaccine Delivery

Effective DNA or mRNA delivery *in vivo* is essential to achieve therapeutic goals. Nucleic acid vaccines have to enter the nucleus or cytoplasm, where transcription or protein expression, respectively, can take place.

As previously mentioned, the greatest challenge of nucleic acid vaccination is low immunogenicity due to cross-membrane barriers. DNA vaccines need to penetrate the nuclear membrane barrier for delivery enhancement, and mRNA vaccines have to cross the lipid-based plasma membrane as efficiently as possible. Hence, several methods to enhance cell delivery and immunogenicity have been developed.

### Physical and Chemical Delivery Methods for Nucleic Acid Vaccines

Similar to conventional protein-based vaccines, nucleic acid vaccines can be delivered by a variety of routes, such as intramuscular (IM), intradermal (ID), mucosal, and transdermal delivery. The failure of nucleic acid vaccines to elicit a strong immune response in humans is due to needle injection delivery. Currently, physical delivery methods using a gene gun or intradermal electroporation may facilitate transportation and enhance immunogenicity (Low et al., 2009; Dupuy et al., 2010, 2011; Vasan et al., 2011; Bagarazzi et al., 2012; Grant-Klein et al., 2012, 2015). Prior studies indicate that applying a gene gun or *in vivo* electroporation for mRNA delivery can induce a strong immune response in mice due to increased mRNA release into the cytoplasm in the context of non-amplifying mRNA or self-amplifying mRNA administration (Qiu et al., 1996; Kofler et al., 2004; Aberle et al., 2005; Steitz et al., 2006; Johansson et al., 2012). In addition, it has been reported that electroporation-enhanced DNA vaccination leads to increased polyfunctional CD8^+^ T cells numbers in patients who received HPV DNA vaccines expressing the E6 and E7 genes of HPV16 and HPV18, respectively (Vasan et al., 2011).

The efficacy of nucleic acid vaccines can be improved significantly by utilizing chemical delivery methods (i.e., nanocarriers). Currently, the components of nanocarriers used in nucleic acid vaccines can be classified into lipid-based nanosystems (Li and Szoka, 2007; Tseng et al., 2009; Gomes-da-Silva et al., 2012; Jayaraman et al., 2012; Sato et al., 2019), polymeric nanomaterials (Tanner et al., 2011; Shim and Kwon, 2012; Dahlman et al., 2014), inorganic nanoparticles (Dahlman et al., 2014; Lin et al., 2016; Shen et al., 2018), and bioinspired nanovehicles (Yoo et al., 2011; Li et al., 2017). On account of the enhanced permeability and retention (EPR) effect, nanotechnology provides a versatile and targeted system for the efficient and safe delivery of nucleic acid vaccines (Pecot et al., 2011; Mura et al., 2013; Xiong et al., 2018). Thus, nanosystems not only protect DNA or mRNA from degradation by enzymes and immune responses, but also boost RNA accumulation in the tumor site (Pecot et al., 2011; Xiong et al., 2018) and thus promote the sustained release of the delivered vaccines (Aral and Akbuga, 2003; Basarkar et al., 2007). Compared with the physical delivery method, the chemical delivery approach provides a new direction for nucleic acid vaccines. Furthermore, different formulations of DNA have been reported, for instance, incorporation of cationic lipids or cholesterol (Donnelly et al., 2005).

### Combination With Vaccine Adjuvants

The use of vaccine adjuvants to increase immunogenicity is a widely acknowledged strategy for nucleic acid vaccines. Vaccine adjuvants play a crucial role through a series of mechanisms, including activation of the innate immune system, induction of the expression of various chemokines, enhancement of antigen uptake and presentation by professional APCs, and upregulation of costimulatory surface molecules expression (Petrovsky and Aguilar, 2004). For instance, alum, as a universal vaccine adjuvant since 1926, contributes to an immune danger signal via the induction of phagocytic cell death. It has been reported that the combination of a *Toxoplasma gondii* DNA vaccine with an alum adjuvant can improve the survival rate of mice (Khosroshahi et al., 2012). In addition, many immunostimulatory molecules encoded by vaccine plasmids, various cytokine genes, and PRR ligands use recombinant DNA technology, which allows them to be co-administered with an antigenic DNA vaccine plasmid to certain cellular compartments or APCs to enhance the immune response (Li and Petrovsky, 2016). For mRNA vaccines, synthetic double-stranded RNA and exogenous RNA extracted from viruses were used as early RNA adjuvants, but severe side effects soon restricted their further application (Field et al., 1967; Absher and Stinebring, 1969). Studies have shown that IVT mRNA can be used as an adjuvant and can be stabilized by compounding or chemical modification (Scheel et al., 2004). In addition, RNA sensor receptors are also effective adjuvant targets. They have evolved to detect and resist viral infections by coordinating the innate and adaptive arms of the immune system (Sadler and Williams, 2008; McCartney and Colonna, 2009). Single and double-stranded RNA molecules are recognized by TLRs 7/8 and 3 in the endosome, respectively (Alexopoulou et al., 2001; Diebold et al., 2004; Heil et al., 2004). However, TLR3 is not only activated by double-stranded RNA, but also activated and transcribed by mRNA released by cells or produced *in vitro* (Karikó et al., 2004). Therefore, the activation of TLR7 and potentially TLR3 is a key adjuvant signal in immune response triggering.

## Nucleic Acid Vaccines for Infectious Diseases and Cancers

Based on the characteristics and immune mechanisms of the two types of nucleic acid vaccines, DNA vaccines are more frequently used for infectious diseases in clinical trials, while mRNA vaccines are more common in cancer research. Recently, when we searched the National Library of Medicine (NIH) for “mRNA vaccine for cancer prevention” clinical trials, a total of 92 results US National Library Of Medicine ClinicalTrials.gov (2020) (2007–2020) were retrieved, and for “mRNA vaccine for prevention of infectious diseases” clinical trials, 11 results (2016–2020) were retrieved. A total of 214 results for DNA vaccines were retrieved, corresponding to 66 results (2007–2020) for cancers and 126 results (2007–2020) for infectious diseases (Medicine). Hence, we summarized the nucleic acid vaccines used to against infectious diseases (Supplementary Table 1) and cancer (Supplementary Table 2) during the search time period.

### Nucleic Acid Vaccines for Infectious Disease Treatment

#### DNA Vaccines

In response to a wide variety of infectious diseases that harm humans, a certain number of DNA vaccines have been developed and have entered the clinical trial stage. However, because some fatal shortcomings have not been resolved, most of the DNA vaccines officially approved for use in the market are aimed at animals for veterinary treatment. For instance, it has been reported that a canarypox vaccination used to treat West Nile virus infection can produce an effective immune response in horses and dogs (Grosenbaugh et al., 2004; Karaca et al., 2005; Dauphin and Zientara, 2007). However, there are currently no DNA vaccines approved for prophylactic human therapy. The first phase I clinical trial of DNA vaccines in humans tested an HIV-1 vaccine candidate in HIV-1-infected people and then in volunteers who were not infected with HIV-1. This study reported that DNA vaccine encoded env and rev genes of HIV was well-tolerate in immunizations and no anti-DNA antibody or adverse reactions were observed. Furthermore, cellular and humoral immune response can be detected by measuring antibody-GMTs against gp120, CTL response and T lymphocyte proliferation both in HIV-1 infected and non-infected people, which indicated this DNA vaccines against HIV-1 was effective (MacGregor et al., 1998). Since then, several institutions have conducted clinical trials of other preventive and therapeutic DNA vaccines, including DNA vaccine trials for influenza, malaria, hepatitis B, and other types of HIV-1 candidate viruses. Although these trials have indicated that the DNA vaccine platform is well tolerated and safe, first-generation DNA vaccines failed to induce a high level of vaccine-specific immunity in humans. Researchers are now focused on designing efficient and safe antiviral vaccines, especially focusing on the development of DNA vaccines against various uncontrolled viral pathogens (i.e., HIV, West Nile virus, and hepatitis C virus), as well as DNA vaccines capable of treating bacteria (i.e., tuberculosis and brucellosis) and protozoan diseases (i.e., leishmaniasis, malaria, and toxoplasmosis) (Marć et al., 2015). This article mainly divides DNA vaccines for infectious disease treatment into three types and reports representative clinical trials to indicate their safety and immunogenicity.

##### Viral DNA vaccines

The viral diseases that are of relatively great concern in clinical trials mainly include AIDS, hepatitis B and C, influenza and warts or cancer caused by human papillomavirus (HPV). Existing research institutions have designed a DNA vaccine for HSV-2, and found that if the DNA vaccine is combined with the adjuvant Vaxfectin (Bal et al., 2012), it can reduce the DNA replication of HSV-2 to a minimal level that is undetectable, while injection without Vaxfectin cannot achieve this. In addition, applying this DNA vaccine with an adjuvant is able to induce higher concentrations of IgG. For HIV-1, the National Institute of Allergy and Infectious Diseases (NIAID) has performed a phase I clinical trial to evaluate the safety and tolerability of the experimental HIV vaccine pGA2/JS2 at two different doses (0.3 and 3.0 mg), which showed that JS2 DNA priming vector was low immunogenicity, good tolerability and safety. Thus, it gives an optimistic prospect for future investigations. For hepatitis B, Maryline Mancini-Bourgine conducted the first clinical trial focused on a hepatitis B vaccine. In recent research, ELISpot technology was used to evaluate the efficacy of an intramuscular DNA vaccine. Ten patients with chronic HBV infection were selected for the trial. The patients were injected intramuscularly three times a day, and response of T cells to the HBV viral antigen was determined with the ELISpot technology. The results showed that after three rounds of injections (three times a day), two patients had T cells in the blood that responded to the viral antigens, and the response rate of specific T cells producing interferon-γ also increased significantly. Quantitative PCR analysis indicated that a decreased serum level of the virus, and no viral infection was found based on a serum test, indicating the effective immune response induced by this vaccine (Karimkhanilouyi and Ghorbian, 2019).

##### Protozoan DNA vaccines

In some tropical regions, protozoan infection is a considerable threat to human survival. A number of investigations have been carried out to find a DNA vaccine against protozoan infections. For example, to combat malaria caused by *Plasmodium falciparum*, a clinical trial evaluated different anti-malarial DNA vaccines. In addition, the European research project LEISHDNAVAX was designed to create and test an effective DNA vaccine against leishmaniasis (Sautter et al., 2011).

Some antimicrobial gene vaccines have been explored in both preclinical trials and clinical trials, and clinical trials focused on *Mycobacterium tuberculosis* have gained increasing attention. Among these trials, a DNA vaccine that expresses the immunogenic *M. tuberculosis* protein Ag85 has been studied in a phase I trial (Smaill and Xing, 2014). Two groups immunized with BCG or not both were safe and well tolerated. Compared with Ad5Ag85A-vaccinated healthy people, previously BCG-immunized people elicited stronger T cell response, which suggested that Ad5Ag85A can be developed as a boost vaccine after BCG priming.

#### RNA Vaccines

Unlike DNA vaccines, RNA vaccines have shown the ability to induce effective neutralizing antibody responses in low-dose immunized animals. The following will summarize the clinical trials on non-amplifying mRNA vaccines and self-amplifying mRNA vaccines used against infectious diseases.

##### Non-amplifying mRNA

Non-amplifying mRNA vaccines can be further divided into dendritic cell mRNA vaccines and directly injected non-amplifying mRNA vaccines delivered by different methods.

(1) Dendritic cell mRNA vaccines.

Infectious disease vaccines developed via DC cells are mainly limited to therapeutic vaccines against HIV-1. A clinical trial in humans evaluated CMV pp65 mRNA-loaded DC vaccination of healthy human volunteers and administration of allogeneic stem cell receptors, and the induction and expansion of the CMV-specific cellular immune response were found (Van Craenenbroeck et al., 2015).

(2) Direct injection of non-amplifying mRNA vaccines.

In an animal trial, researchers used the protamine-based RNActive platform that encodes the rabies virus glycoprotein for immunization. The results showed that in mice, the vaccine was able to induce protective immunity against rabies virus, which can cause fatal inflammation in the brain of mice and produce an effective neutralizing antibody response in pigs (Schnee et al., 2016). Other infectious disease vaccines have successfully used lipid or polymer delivery systems. For example, PEI-complexed mRNA can be effectively delivered to mice, thereby separately inducing an HIV-1-specific immune response. Kranz et al. (2016) also used the lipid-complexed mRNA to encode influenza virus HA to immunize mice intravenously. Both studies showed excellent immune activity. Direct injection of non-amplifying mRNA vaccines is an attractive form of vaccination, because the delivery method is simple and economical compared to other methods in the case of limited resources.

##### Self-amplifying mRNA

A study found that with a prime-boost immunization regimen, viral delivery of self-amplifying mRNA proved that factors such as timing and dose affect the phenotype of the immunization-induced memory T cell population by characterizing the intensity and cellular phenotype of the CD8^+^ T cell response (Knudsen et al., 2014). Another study found that protective immune responses occurred in mouse and ferret animal models after vaccination with self-amplifying mRNA encoding influenza virus haemagglutinin, which proved that vaccine-specific antibodies play a crucial role in protecting against homologous influenza virus strains. Additionally, vaccine-specific T cells were also found to support the control of heterotypic infections (Brazzoli et al., 2016). Self-amplifying mRNA, encoding influenza antigens can be modified with chitosan-containing LNP or polyethyleneimine (PEI), which can elicit T and B cell immune responses in mice after subcutaneous injection (McCullough et al., 2014; Démoulins et al., 2016). Chahal et al. (2016) confirmed that vaccination with a self-amplifying mRNA encoding Zika virus prM-E could produce antigens recognized by mouse-specific antibodies and trigger CD8^+^ T cell responses by using an LNPs89 delivery platform consisting of chemically modified ionizable dendritic cell complexes.

Currently, the novel coronavirus, SARS-CoV-2, have spread rapidly since December 2019 and gained the attention of researchers worldwide due to its strong infectivity and lethality rate. The development of a vaccine against the new coronavirus is urgently needed. A large number of vaccines against the new coronavirus have entered clinical trials. Multiple strategies are applied to produce vaccines against COVID-19. The commonest is exposed spike (S) glycoprotein or S protein which serves as the main trigger of neutralizing antibody, such as full-length S protein or S1 receptor binding domain (RBD) and expressing in virus-like particles (VLP), DNA or viral vector (Graham et al., 2013). Moderna developed a novel lipid nanoparticle (LNP)-encapsulated mRNA-based vaccine, mRNA-1273, which encoded the S protein of SARS-CoV-2. Recent studies reported that mRNA-1273 vaccines showed 94.1% efficacy in preventing COVID-19 and no severe adverse reactions except for local and systemic reactions (Baden et al., 2020), which indicated that mRNA vaccines provided a effective and stable platform in infectious diseases treatments.

### Nucleic Acid Vaccines for Cancer Treatment

#### DNA Vaccines

One of the earliest clinical trials on cancer treatment using DNA vaccines was conducted in 1998 (MacGregor et al., 1998). This trial, specific for prostate cancer, used a prostate membrane antigen as the cancer antigen, as well as adenoviral vectors and GM-CSF as an adjuvant. Since then, the results of a certain number of clinical trials have indicated that DNA vaccines used to treat cancer are well tolerated and do not cause severe adverse reactions. Additionally, the cost-effectiveness of DNA vaccines is considerable, and thus repeated vaccination can be performed for long-term protection (Yang et al., 2014). The DNA vaccines that have entered the clinical trial stage for several cancers will be described below.

##### Breast cancer

The Mammaglobin-A (Mam-A) cDNA vaccine can induce antitumour- immunity in breast cancer. The vaccine encoding Mam-A cDNA can generate a Mam-A-specific CD8^+^ T cell immune response. A phase I clinical trial evaluating the Mam-A cDNA vaccine (Tiriveedhi et al., 2013) observed that the vaccine had immune activity in patients with stage 4 metastatic breast cancer. Some research institutions have conducted phase I/II clinical trials using DNA vaccines encoding human prostatic acid phosphatase (PAP). In one trial, the subjects were 22 patients with biochemical recurrence of prostate cancer. After receiving treatment, three patients developed papi-specific IFNgC CD8^+^ T cells, and nine patients exhibited proliferation of papi-specific CD4^+^ and CD8^+^ T cells, which suggested that the DNA vaccine had biological activity that could induce an antitumor immune response. In addition, no obvious adverse reactions occurred after vaccination, and the serum prostate-specific antigen doubling time (PSA DT) of several patients increased. These results illustrated the safety of the DNA vaccine and suggested that it might have an impact on the tumor growth rate (McNeel et al., 2009).

##### Cervical cancer

In a phase I clinical trial, the highly optimized DNA vaccine VGX-3100, encoding the human papillomavirus (HPV) 16 and 18 E6/E7 antigens, was tested. This trial evaluated 18 postresection patients with grade 2/3 cervical intraepithelial neoplasia (CIN) who received a three-dose regimen (0.6, 2, or 6 mg of DNA per dose) via three intramuscular injections. An IFN-γ ELISpot assay found that 78% of subjects had an increased T helper 1 (T_*H*_1)-biased cellular immune response. In the different dose groups, the average peak value of the T cell response induced by VGX-3100 was 642 to 1458 SFU per 106 PBMCs. The results showed that the vaccine induced strong and extensive cellular immunity. In addition, no serious adverse reactions, grade 3/4 adverse reactions or injection site reactions were observed, which showed the safety of VGX-3100 (Bagarazzi et al., 2012).

#### RNA Vaccines

*In vitro*, DC loading is an essential method for generating cellular immunity in organisms to fight cancer. Boczkowski et al. (1996) reported for the first time that electroporation of dendritic cells with mRNA could induce an effective immune response against tumor antigens. Since then, a variety of immunomodulatory proteins have been identified, and they exist in the form of mRNA-encoded adjuvants, which can enhance the immune activity of DC cancer vaccines. At present, many clinical trials of DC vaccines against various cancer types have been conducted, such as vaccines for metastatic prostate cancer, brain cancer, metastatic lung cancer, acute myeloid leukemia, renal cell carcinoma, melanoma, and pancreatic cancer (Van Lint et al., 2015). A new research direction has emerged for vaccine development. It is the combination of electroporation of mRNA into dendritic cells with conventional cemotherapeutic drugs or immune checkpoint inhibitors. In a clinical trial, patients with stage III or stage IV melanoma were treated with a mixture that contained ipilimumab, which is an anti-CTL antigen 4 (CTLA4) monoclonal antibody, DCs and an adjuvant TriMix mRNA encoding three immune-activating proteins: CD70, CD40 ligand (CD40L), and constitutively active TLR4 (Wilgenhof et al., 2016). The mixture can be combined with antigen-encoded mRNA or mRNAs132 for treatment via electroporation. The data used to determine the 6-month disease control rate (51%) and the overall tumor response rate (38%) showed highly durable antitumour activity, which suggested that the overall survival of patients with advanced melanoma was improved.

The route and method of administration of mRNA vaccines will have significant influences on the immune effect. A variety of formats of mRNA cancer vaccines have been developed using traditional (e.g., subcutaneous, intradermal, and intramuscular) and non-traditional (e.g., intranasal, intravenous, and intratumoural) routes. Several studies have shown that naked mRNA injected intranasally can be selectively absorbed by DCs, thereby causing an effective prophylactic or therapeutic antitumor T cell response (Bialkowski et al., 2016). In prior research, patients with metastatic melanoma received DC electroporation by intranasal administration of mRNA encoding melanoma-associated TAA (tyrosinase and gp100) together with TriMix mRNA encoding CD40L, CD70, and caTLR4 (constitutively active TLR4). The results proved the safety of this method. However, the method induced limited antitumour responses against tyrosinase and gp100 (Bol et al., 2015). Therefore, the efficacy of mRNA vaccines can be enhanced via various administration methods and combinations with chemotherapy or other antitumor therapies.

## Safety and Prospects of Nucleic Acid Vaccines in Clinical Practice

People have long been concerned about the safety of DNA vaccines, mainly due to the stable integration of transfected DNA into the genome of somatic or germinal cells, which may lead to dysregulation of gene expression and gene mutation. The overall safety of DNA vaccine has been fully demonstrated in several clinical trials, and adverse reactions are limited to local reactions at the injection site (Fioretti et al., 2014). Therefore, the main research direction for clinical trials of DNA vaccines has become proving their effectiveness.

Although clinical trials using DNA vaccines have found that these vaccines can effectively induce cellular and humoural responses, the strength of these reactions is usually not sufficient to produce significant clinical benefit. Additionally, due to tumor immune tolerance to endogenous autoantigens, DNA vaccines still need to be improved in regard to inducing effective antigen-specific cellular immune responses. Therefore, the development of a method to bypass immune tolerance is warranted in DNA vaccine development. In addition, DNA vaccines can be used in combination with other cancer treatments to further fight against tumors (Yang et al., 2014).

Because there are no toxic chemicals involved in the production of mRNA, and the environment of the platform is clean and free of foreign virus contamination, the production risk of mRNA is significantly lower than that of other vaccine platforms (i.e., live virus, viral vector, subunit protein vaccine, and inactivated virus). After vaccination, the theoretical risk of infection and integration of the vector into host cells will not exist, unlike in DNA vaccination (Pardi et al., 2018). In short, mRNA vaccines are a relatively safe form. There have been clinical trials studying several different mRNA vaccines from phase I to phase IIb, and the results of these studies have shown their safety and tolerability (Supplementary Table 1). However, recent human trials have shown that different mRNA platforms show different degrees of adverse reactions at the injection site or throughout the body after vaccination (Bahl et al., 2017). mRNA vaccines based on some platforms can induce potent type I interferon responses, which may be related to inflammation and autoimmunity (Abd El-Aziz and Stockand, 2020). Another safety issue is related to extracellular RNA, which can increase the permeability of endothelial cells, resulting in oedema (Fischer et al., 2007). Therefore, the instability of RNA itself and the presence of other physiological obstacles inhibit the transmission and transfection of RNA, hindering its clinical application in cancer treatment (Rosenblum et al., 2018). In addition, exogenous RNA may be cleared by the body’s own immune system. However, the preparation of the mRNA candidate vaccines evaluated in clinical trials has not been involved a delivery system, which indicates the need to further improve the delivery system for mRNA vaccines (Wadhwa et al., 2020). To overcome these obstacles and ensure the safe delivery of RNA therapeutics to target sites, a nanoparticle-based delivery system has been explored as a potential RNA delivery tool for preclinical applications (Chen et al., 2017). This technology has been successful in preclinical research, followed by clinical trials of cancer immunotherapy with different forms of RNA-mediated nano-delivery systems (Lin et al., 2020).

## Conclusion and Perspectives

Currently, nucleic acid vaccines are undergoing rapid development for the treatment of infectious diseases and cancers. Pandemics and malignant tumors, such as HIV, AIDS, Ebola, COVID-19, breast cancer, and melanoma, have raised people’s awareness of the global threats to human health and promoted the development of nucleic acid vaccine platforms, enabling researchers to cope with the challenges of severe situations. The dozens of preclinical and clinical studies indicate that nucleic acid vaccines not only have a significant efficacy in the treatment of infectious diseases, but also have demonstrated potency in cancer applications. Although nucleic acid vaccines provide a certain number of advantages over conventional vaccines, further optimization is necessary before they become the main treatment strategy for human patients. This article summarizes the optimization strategies based on the shortcomings of nucleic acid vaccines from three perspectives: physical methods (i.e., a gene gun or electroporation), chemical methods (i.e., lipid or polymer-based nanocarriers), and adjuvants. Therefore, the uptake and membrane-penetrating capacity of nucleic acid vaccines can be promoted, resulting in a stronger immune response. An increasing number of molecular adjuvants, such as adhesion molecules, cytokines, chemokines, and transcription factors, are undergoing safety and tolerability assessment. Similarly, the continuous development of vaccine delivery methods is promising and worthy of further research. While it seems unlikely that a technology that can provide a suitable solution for every single patient will be developed, combining existing technology and constantly improving the understanding of human immunology will provide better tools to fight known and emerging global threats.

## Author Contributions

All authors read and approved the final manuscript. FQ wrote the initial manuscript. FX contributed new ideas and created the figures. HC and BC created Supplementary Tables 1, 2, respectively. YF and PZ contributed to writing materials. ML and JC revised the manuscript and approved the final version.

## Conflict of Interest

The authors declare that the research was conducted in the absence of any commercial or financial relationships that could be construed as a potential conflict of interest.

## References

[B1] Abd El-Aziz T. M. Stockand J. D. (2020). Recent progress and challenges in drug development against COVID-19 coronavirus (SARS-CoV-2) - an update on the status. *Infect. Genet. Evol.* 83:104327. 10.1016/j.meegid.2020.104327 32320825 PMC7166307

[B2] Aberle J. H. Aberle S. W. Kofler R. M. Mandl C. W. (2005). Humoral and cellular immune response to RNA immunization with flavivirus replicons derived from tick-borne encephalitis virus. *J. Virol.* 79 15107–15113. 10.1128/jvi.79.24.15107-15113.2005 16306582 PMC1316042

[B3] Absher M. Stinebring W. R. (1969). Toxic properties of a synthetic double-stranded RNA. Endotoxin-like properties of poly I. poly C, an interferon stimulator. *Nature* 223 715–717. 10.1038/223715a0 5805520

[B4] Albert M. L. Sauter B. Bhardwaj N. (1998). Dendritic cells acquire antigen from apoptotic cells and induce class I-restricted CTLs. *Nature* 392 86–89. 10.1038/32183 9510252

[B5] Alexopoulou L. Holt A. C. Medzhitov R. Flavell R. A. (2001). Recognition of double-stranded RNA and activation of NF-kappaB by Toll-like receptor 3. *Nature* 413 732–738. 10.1038/35099560 11607032

[B6] Aral C. Akbuga J. (2003). Preparation and in vitro transfection efficiency of chitosan microspheres containing plasmid DNA:poly(L-lysine) complexes. *J. Pharm. Sci.* 6 321–326. 14738712

[B7] Baden L. R. El Sahly H. M. Essink B. Kotloff K. Frey S. Novak R. (2020). Efficacy and Safety of the mRNA-1273 SARS-CoV-2 Vaccine. *N. Engl. J. Med*. 384 403–416. 10.1056/NEJMoa2035389 33378609 PMC7787219

[B8] Bagarazzi M. L. Yan J. Morrow M. P. Shen X. Parker R. L. Lee J. C. (2012). Immunotherapy against HPV16/18 generates potent TH1 and cytotoxic cellular immune responses. *Sci. Transl. Med.* 4:155ra138. 10.1126/scitranslmed.3004414 23052295 PMC4317299

[B9] Bahl K. Senn J. J. Yuzhakov O. Bulychev A. Brito L. A. Hassett K. J. (2017). Preclinical and Clinical Demonstration of Immunogenicity by mRNA Vaccines against H10N8 and H7N9 Influenza Viruses. *Mol. Ther.* 25 1316–1327. 10.1016/j.ymthe.2017.03.035 28457665 PMC5475249

[B10] Bai H. Lester G. M. S. Petishnok L. C. Dean D. A. (2017). Cytoplasmic transport and nuclear import of plasmid DNA. *Biosci. Rep.* 37: BSR20160616. 10.1042/bsr20160616 29054961 PMC5705778

[B11] Bal S. M. Slütter B. Verheul R. Bouwstra J. A. Jiskoot W. (2012). Adjuvanted, antigen loaded N-trimethyl chitosan nanoparticles for nasal and intradermal vaccination: adjuvant- and site-dependent immunogenicity in mice. *Eur. J. Pharm. Sci.* 45 475–481. 10.1016/j.ejps.2011.10.003 22009113

[B12] Banerjee A. K. (1980). 5’-terminal cap structure in eucaryotic messenger ribonucleic acids. *Microbiol. Rev.* 44 175–205. 10.1128/mr.44.2.175-205.1980 6247631 PMC373176

[B13] Basarkar A. Devineni D. Palaniappan R. Singh J. (2007). Preparation, characterization, cytotoxicity and transfection efficiency of poly(DL-lactide-co-glycolide) and poly(DL-lactic acid) cationic nanoparticles for controlled delivery of plasmid DNA. *Int. J. Pharm.* 343 247–254. 10.1016/j.ijpharm.2007.05.023 17611054 PMC6186392

[B14] Bauer M. Redecke V. Ellwart J. W. Scherer B. Kremer J. P. Wagner H. (2001). Bacterial CpG-DNA triggers activation and maturation of human CD11c-, CD123+ dendritic cells. *J. Immunol.* 166 5000–5007. 10.4049/jimmunol.166.8.5000 11290780

[B15] Bialkowski L. van Weijnen A. Van der Jeught K. Renmans D. Daszkiewicz L. Heirman C. (2016). Intralymphatic mRNA vaccine induces CD8 T-cell responses that inhibit the growth of mucosally located tumours. *Sci. Rep.* 6:22509. 10.1038/srep22509 26931556 PMC4773884

[B16] Boczkowski D. Nair S. K. Snyder D. Gilboa E. (1996). Dendritic cells pulsed with RNA are potent antigen-presenting cells in vitro and in vivo. *J. Exp. Med.* 184 465–472. 10.1084/jem.184.2.465 8760800 PMC2192710

[B17] Bol K. F. Figdor C. G. Aarntzen E. H. Welzen M. E. van Rossum M. M. Blokx W. A. (2015). Intranodal vaccination with mRNA-optimized dendritic cells in metastatic melanoma patients. *Oncoimmunology* 4:e1019197. 10.1080/2162402x.2015.1019197 26405571 PMC4570143

[B18] Brazzoli M. Magini D. Bonci A. Buccato S. Giovani C. Kratzer R. (2016). Induction of Broad-Based Immunity and Protective Efficacy by Self-amplifying mRNA Vaccines Encoding Influenza Virus Hemagglutinin. *J. Virol.* 90 332–344. 10.1128/jvi.01786-15 26468547 PMC4702536

[B19] Chahal J. S. Khan O. F. Cooper C. L. McPartlan J. S. Tsosie J. K. Tilley L. D. (2016). Dendrimer-RNA nanoparticles generate protective immunity against lethal Ebola, H1N1 influenza, and Toxoplasma gondii challenges with a single dose. *Proc. Natl. Acad. Sci. U. S. A.* 113 E4133–E4142. 10.1073/pnas.1600299113 27382155 PMC4961123

[B20] Chen B. Dai W. He B. Zhang H. Wang X. Wang Y. (2017). Current Multistage Drug Delivery Systems Based on the Tumor Microenvironment. *Theranostics* 7 538–558. 10.7150/thno.16684 28255348 PMC5327631

[B21] Chen Z. Y. He C. Y. Ehrhardt A. Kay M. A. (2003). Minicircle DNA vectors devoid of bacterial DNA result in persistent and high-level transgene expression in vivo. *Mol. Ther.* 8 495–500. 10.1016/s1525-0016(03)00168-0 12946323

[B22] Colluru V. T. Zahm C. D. McNeel D. G. (2016). Mini-intronic plasmid vaccination elicits tolerant LAG3(+) CD8(+) T cells and inferior antitumor responses. *Oncoimmunology* 5:e1223002. 10.1080/2162402x.2016.1223002 27853647 PMC5087309

[B23] Dahlman J. E. Barnes C. Khan O. Thiriot A. Jhunjunwala S. Shaw T. E. (2014). In vivo endothelial siRNA delivery using polymeric nanoparticles with low molecular weight. *Nat. Nanotechnol.* 9 648–655. 10.1038/nnano.2014.84 24813696 PMC4207430

[B24] Dauphin G. Zientara S. (2007). West Nile virus: recent trends in diagnosis and vaccine development. *Vaccine* 25 5563–5576. 10.1016/j.vaccine.2006.12.005 17292514

[B25] Démoulins T. Milona P. Englezou P. C. Ebensen T. Schulze K. Suter R. (2016). Polyethylenimine-based polyplex delivery of self-replicating RNA vaccines. *Nanomedicine* 12 711–722. 10.1016/j.nano.2015.11.001 26592962

[B26] Diebold S. S. Kaisho T. Hemmi H. Akira S. Reise Sousa C. (2004). Innate antiviral responses by means of TLR7-mediated recognition of single-stranded RNA. *Science* 303 1529–1531. 10.1126/science.1093616 14976261

[B27] Dominski Z. Marzluff W. F. (1999). Formation of the 3’ end of histone mRNA. *Gene* 239 1–14. 10.1016/s0378-1119(99)00367-4 10571029

[B28] Donnelly J. J. Douglas M. Jansen K. U. Ellis R. W. Montgomery D. L. Liu M. A. (1996). Protection against Papillomavirus with a Polynucleotide Vaccine. *J. Infect. Dis.* 173 314–20. 10.1093/infdis/173.2.314 8568291

[B29] Donnelly J. J. Wahren B. Liu M. A. (2005). DNA vaccines: progress and challenges. *J. Immunol.* 175 633–639. 10.4049/jimmunol.175.2.633 16002657

[B30] Dupuy L. C. Richards M. J. Ellefsen B. Chau L. Luxembourg A. Hannaman D. (2011). A DNA Vaccine for Venezuelan Equine Encephalitis Virus Delivered by Intramuscular Electroporation Elicits High Levels of Neutralizing Antibodies in Multiple Animal Models and Provides Protective Immunity to Mice and Nonhuman Primates. *Clin. Vacc. Immunol.* 18 707–716. 10.1128/cvi.00030-11 21450977 PMC3122536

[B31] Dupuy L. C. Richards M. J. Reed D. S. Schmaljohn C. S. (2010). Immunogenicity and protective efficacy of a DNA vaccine against Venezuelan equine encephalitis virus aerosol challenge in nonhuman primates. *Vaccine* 28 7345–7350. 10.1016/j.vaccine.2010.09.005 20851089

[B32] Field A. K. Tytell A. A. Lampson G. P. Hilleman M. R. (1967). Inducers of interferon and host resistance. II. Multistranded synthetic polynucleotide complexes. *Proc. Natl. Acad. Sci. U. S. A.* 58 1004–1010. 10.1073/pnas.58.3.1004 5233831 PMC335739

[B33] Fioretti D. Iurescia S. Rinaldi M. (2014). Recent advances in design of immunogenic and effective naked DNA vaccines against cancer. *Recent Pat. Antic. Drug Discov.* 9 66–82. 10.2174/1574891x113089990037 23444943

[B34] Fischer S. Gerriets T. Wessels C. Walberer M. Kostin S. Stolz E. (2007). Extracellular RNA mediates endothelial-cell permeability via vascular endothelial growth factor. *Blood* 110 2457–2465. 10.1182/blood-2006-08-040691 17576819

[B35] Fuller D. Haynes J. (1994). A qualitative progression in HIV type 1 glycoprotein 120-specific cytotoxic cellular and humoral immune responses in mice receiving a DNA-based glycoprotein 120 vaccine. *AIDS Res. Hum. Retrovir.* 10 1433–1441. 10.1089/aid.1994.10.1433 7888198

[B36] Gallie D. R. (1991). The cap and poly(A) tail function synergistically to regulate mRNA translational efficiency. *Genes Dev.* 5 2108–2116. 10.1101/gad.5.11.2108 1682219

[B37] Gomes-da-Silva L. C. Fonseca N. A. Moura V. Pedroso, de Lima M. C. Simões S. (2012). Lipid-based nanoparticles for siRNA delivery in cancer therapy: paradigms and challenges. *Acc. Chem. Res.* 45 1163–1171. 10.1021/ar300048p 22568781

[B38] Graham R. L. Donaldson E. F. Baric R. S. (2013). A decade after SARS: strategies for controlling emerging coronaviruses. *Nat. Rev. Microbiol.* 11 836–848. 10.1038/nrmicro3143 24217413 PMC5147543

[B39] Grant-Klein R. J. Altamura L. A. Badger C. V. Bounds C. E. Van Deusen N. M. Kwilas S. A. (2015). Codon-optimized filovirus DNA vaccines delivered by intramuscular electroporation protect cynomolgus macaques from lethal Ebola and Marburg virus challenges. *Hum. Vacc. Immunother.* 11 1991–2004. 10.1080/21645515.2015.1039757 25996997 PMC4635690

[B40] Grant-Klein R. J. Van Deusen N. M. Badger C. V. Hannaman D. Dupuy L. C. Schmaljohn C. S. (2012). A multiagent filovirus DNA vaccine delivered by intramuscular electroporation completely protects mice from ebola and Marburg virus challenge. *Hum. Vacc. Immunother.* 8 1703–1706. 10.4161/hv.21873 22922764 PMC3601145

[B41] Grosenbaugh D. A. Backus C. S. Karaca K. Minke J. M. Nordgren R. M. (2004). The anamnestic serologic response to vaccination with a canarypox virus-vectored recombinant West Nile virus (WNV) vaccine in horses previously vaccinated with an inactivated WNV vaccine. *Vet. Ther.* 5 251–257. 15719324

[B42] Groux H. (2003). Type 1 T-regulatory cells: their role in the control of immune responses. *Transplantation* 75 8s–12s. 10.1097/01.Tp.0000067944.90241.Bd 12819483

[B43] Hartmann G. Weiner G. J. Krieg A. M. (1999). CpG DNA: a potent signal for growth, activation, and maturation of human dendritic cells. *Proc. Natl. Acad. Sci. U. S. A.* 96 9305–9310. 10.1073/pnas.96.16.9305 10430938 PMC17777

[B44] Heil F. Hemmi H. Hochrein H. Ampenberger F. Kirschning C. Akira S. (2004). Species-specific recognition of single-stranded RNA via toll-like receptor 7 and 8. *Science* 303 1526–1529. 10.1126/science.1093620 14976262

[B45] Hemmi H. Takeuchi O. Kawai T. Kaisho T. Sato S. Sanjo H. (2000). A Toll-like receptor recognizes bacterial DNA. *Nature* 408 740–745. 10.1038/35047123 11130078

[B46] Horwitz D. A. Zheng S. G. Gray J. D. (2003). The role of the combination of IL-2 and TGF-beta or IL-10 in the generation and function of CD4+ CD25+ and CD8+ regulatory T cell subsets. *J. Leukoc. Biol.* 74 471–478. 10.1189/jlb.0503228 14519757 PMC7166542

[B47] Jayaraman M. Ansell S. M. Mui B. L. Tam Y. K. Chen J. Du X. (2012). Maximizing the potency of siRNA lipid nanoparticles for hepatic gene silencing in vivo. *Angew. Chem. Int. Ed. Engl.* 51 8529–8533. 10.1002/anie.201203263 22782619 PMC3470698

[B48] Johansson D. X. Ljungberg K. Kakoulidou M. Liljeström P. (2012). Intradermal electroporation of naked replicon RNA elicits strong immune responses. *PLoS One* 7:e29732. 10.1371/journal.pone.0029732 22238645 PMC3251598

[B49] Kadowaki N. Antonenko S. Liu Y. J. (2001). Distinct CpG DNA and polyinosinic-polycytidylic acid double-stranded RNA, respectively, stimulate CD11c- type 2 dendritic cell precursors and CD11c+ dendritic cells to produce type I IFN. *J. Immunol.* 166 2291–2295. 10.4049/jimmunol.166.4.2291 11160284

[B50] Karaca K. Bowen R. Austgen L. E. Teehee M. Siger L. Grosenbaugh D. (2005). Recombinant canarypox vectored West Nile virus (WNV) vaccine protects dogs and cats against a mosquito WNV challenge. *Vaccine* 23 3808–3813. 10.1016/j.vaccine.2005.02.020 15893618

[B51] Karikó K. Muramatsu H. Ludwig J. Weissman D. (2011). Generating the optimal mRNA for therapy: HPLC purification eliminates immune activation and improves translation of nucleoside-modified, protein-encoding mRNA. *Nucleic Acids Res.* 39:e142. 10.1093/nar/gkr695 21890902 PMC3241667

[B52] Karikó K. Muramatsu H. Welsh F. A. Ludwig J. Kato H. Akira S. (2008). Incorporation of pseudouridine into mRNA yields superior nonimmunogenic vector with increased translational capacity and biological stability. *Mol. Ther.* 16 1833–1840. 10.1038/mt.2008.200 18797453 PMC2775451

[B53] Karikó K. Ni H. Capodici J. Lamphier M. Weissman D. (2004). mRNA is an endogenous ligand for Toll-like receptor 3. *J. Biol. Chem.* 279 12542–12550. 10.1074/jbc.M310175200 14729660

[B54] Karimkhanilouyi S. Ghorbian S. (2019). Nucleic acid vaccines for hepatitis B and C virus. *Infect. Genet. Evol.* 75:103968. 10.1016/j.meegid.2019.103968 31325609

[B55] Kay M. A. He C. Y. Chen Z. Y. (2010). A robust system for production of minicircle DNA vectors. *Nat. Biotechnol.* 28 1287–1289. 10.1038/nbt.1708 21102455 PMC4144359

[B56] Khosroshahi K. H. Ghaffarifar F. Sharifi Z. D’Souza S. Dalimi A. Hassan Z. M. (2012). Comparing the effect of IL-12 genetic adjuvant and alum non-genetic adjuvant on the efficiency of the cocktail DNA vaccine containing plasmids encoding SAG-1 and ROP-2 of Toxoplasma gondii. *Parasitol. Res.* 111 403–411. 10.1007/s00436-012-2852-7 22350714

[B57] Knudsen M. L. Ljungberg K. Kakoulidou M. Kostic L. Hallengärd D. García-Arriaza J. (2014). Kinetic and phenotypic analysis of CD8+ T cell responses after priming with alphavirus replicons and homologous or heterologous booster immunizations. *J. Virol.* 88 12438–12451. 10.1128/jvi.02223-14 25122792 PMC4248943

[B58] Koff W. C. Burton D. R. Johnson P. R. Walker B. D. King C. R. Nabel G. J. (2013). Accelerating next-generation vaccine development for global disease prevention. *Science* 340:1232910. 10.1126/science.1232910 23723240 PMC4026248

[B59] Kofler R. M. Aberle J. H. Aberle S. W. Allison S. L. Heinz F. X. Mandl C. W. (2004). Mimicking live flavivirus immunization with a noninfectious RNA vaccine. *Proc. Natl. Acad. Sci. U. S. A.* 101 1951–1956. 10.1073/pnas.0307145101 14769933 PMC357033

[B60] Kranz L. M. Diken M. Haas H. Kreiter S. Loquai C. Reuter K. C. (2016). Systemic RNA delivery to dendritic cells exploits antiviral defence for cancer immunotherapy. *Nature* 534 396–401. 10.1038/nature18300 27281205

[B61] Kutzler M. A. Weiner D. B. (2008). DNA vaccines: ready for prime time? *Nat. Rev. Genet.* 9 776–788. 10.1038/nrg2432 18781156 PMC4317294

[B62] Leitner W. W. Hammerl P. Thalhamer J. (2001). Nucleic acid for the treatment of cancer: genetic vaccines and DNA adjuvants. *Curr. Pharm. Des.* 7 1641–1667. 10.2174/1381612013397249 11562304

[B63] Li L. Petrovsky N. (2016). Molecular mechanisms for enhanced DNA vaccine immunogenicity. *Exp. Rev. Vacc.* 15 313–329. 10.1586/14760584.2016.1124762 26707950 PMC4955855

[B64] Li W. Szoka F. C. Jr. (2007). Lipid-based nanoparticles for nucleic acid delivery. *Pharm. Res.* 24 438–449. 10.1007/s11095-006-9180-5 17252188

[B65] Li X. Liang Q. Zhang W. Li Y. Ye J. Zhao F. (2017). Bio-inspired bioactive glasses for efficient microRNA and drug delivery. *J. Mater Chem. B* 5 6376–6384. 10.1039/c7tb01021d 32264454

[B66] Lilic D. Ghosh S. K. (1994). Liver dysfunction and DNA antibodies after hepatitis B vaccination. *Lancet* 344 1292–1293. 10.1016/s0140-6736(94)90776-5 7967997

[B67] Lin G. Mi P. Chu C. Zhang J. Liu G. (2016). Inorganic Nanocarriers Overcoming Multidrug Resistance for Cancer Theranostics. *Adv. Sci.* 3:1600134. 10.1002/advs.201600134 27980988 PMC5102675

[B68] Lin Y. X. Wang Y. Blake S. Yu M. Mei L. Wang H. (2020). RNA Nanotechnology-Mediated Cancer Immunotherapy. *Theranostics* 10 281–299. 10.7150/thno.35568 31903120 PMC6929632

[B69] Low L. Mander A. McCann K. Dearnaley D. Tjelle T. Mathiesen I. (2009). DNA vaccination with electroporation induces increased antibody responses in patients with prostate cancer. *Hum. Gene. Ther.* 20 1269–1278. 10.1089/hum.2009.067 19619001

[B70] MacGregor R. R. Boyer J. D. Ugen K. E. Lacy K. E. Gluckman S. J. Bagarazzi M. L. (1998). First human trial of a DNA-based vaccine for treatment of human immunodeficiency virus type 1 infection: safety and host response. *J. Infect. Dis.* 178 92–100. 10.1086/515613 9652427

[B71] Marć M. A. Domínguez-Álvarez E. Gamazo C. (2015). Nucleic acid vaccination strategies against infectious diseases. *Exp. Opin. Drug. Deliv.* 12 1851–1865. 10.1517/17425247.2015.1077559 26365499

[B72] Maruggi G. Zhang C. Li J. Ulmer J. B. Yu D. (2019). mRNA as a Transformative Technology for Vaccine Development to Control Infectious Diseases. *Mol. Ther.* 27 757–772. 10.1016/j.ymthe.2019.01.020 30803823 PMC6453507

[B73] McCartney S. A. Colonna M. (2009). Viral sensors: diversity in pathogen recognition. *Immunol. Rev.* 227 87–94. 10.1111/j.1600-065X.2008.00726.x 19120478 PMC7165970

[B74] McCullough K. C. Bassi I. Milona P. Suter R. Thomann-Harwood L. Englezou P. (2014). Self-replicating Replicon-RNA Delivery to Dendritic Cells by Chitosan-nanoparticles for Translation In Vitro and In Vivo. *Mol. Ther. Nucleic Acids* 3:e173. 10.1038/mtna.2014.24 25004099 PMC4121514

[B75] McNeel D. G. Dunphy E. J. Davies J. G. Frye T. P. Johnson L. E. Staab M. J. (2009). Safety and immunological efficacy of a DNA vaccine encoding prostatic acid phosphatase in patients with stage D0 prostate cancer. *J. Clin. Oncol.* 27 4047–4054. 10.1200/jco.2008.19.9968 19636017 PMC2734418

[B76] Medzhitov R. (2007). Recognition of microorganisms and activation of the immune response. *Nature* 449 819–826. 10.1038/nature06246 17943118

[B77] Miliotou A. N. Papadopoulou L. C. (2020). In Vitro-Transcribed (IVT)-mRNA CAR Therapy Development. *Methods Mol. Biol.* 2086 87–117. 10.1007/978-1-0716-0146-4_7 31707670

[B78] Monjezi R. Miskey C. Gogishvili T. Schleef M. Schmeer M. Einsele H. (2017). Enhanced CAR T-cell engineering using non-viral Sleeping Beauty transposition from minicircle vectors. *Leukemia* 31 186–194. 10.1038/leu.2016.180 27491640

[B79] Mor G. Singla M. Steinberg A. D. Hoffman S. L. Okuda K. Klinman D. M. (1997). Do DNA vaccines induce autoimmune disease? *Hum. Gene Ther.* 8 293–300. 10.1089/hum.1997.8.3-293 9048196

[B80] Morse M. A. Clay T. M. Mosca P. Lyerly H. K. (2002). Immunoregulatory T cells in cancer immunotherapy. *Exp. Opin. Biol. Ther.* 2 827–834. 10.1517/14712598.2.8.827 12517262

[B81] Moseman E. A. Liang X. Dawson A. J. Panoskaltsis-Mortari A. Krieg A. M. Liu Y. J. (2004). Human plasmacytoid dendritic cells activated by CpG oligodeoxynucleotides induce the generation of CD4+CD25+ regulatory T cells. *J. Immunol.* 173 4433–4442. 10.4049/jimmunol.173.7.4433 15383574

[B82] Munroe D. Jacobson A. (1990). mRNA poly(A) tail, a 3’ enhancer of translational initiation. *Mol. Cell Biol.* 10 3441–3455. 10.1128/mcb.10.7.3441 1972543 PMC360780

[B83] Munye M. M. Tagalakis A. D. Barnes J. L. Brown R. E. McAnulty R. J. Howe S. J. (2016). Minicircle DNA Provides Enhanced and Prolonged Transgene Expression Following Airway Gene Transfer. *Sci. Rep.* 6:23125. 10.1038/srep23125 26975732 PMC4792149

[B84] Mura S. Nicolas J. Couvreur P. (2013). Stimuli-responsive nanocarriers for drug delivery. *Nat. Mater.* 12 991–1003. 10.1038/nmat3776 24150417

[B85] Nishikawa H. Sakaguchi S. (2010). Regulatory T cells in tumor immunity. *Int. J. Cancer* 127 759–767. 10.1002/ijc.25429 20518016

[B86] Pardi N. Hogan M. J. Porter F. W. Weissman D. (2018). mRNA vaccines - a new era in vaccinology. *Nat. Rev. Drug Discov.* 17 261–279. 10.1038/nrd.2017.243 29326426 PMC5906799

[B87] Pardoll D. (2003). Does the immune system see tumors as foreign or self? *Annu. Rev. Immunol.* 21 807–839. 10.1146/annurev.immunol.21.120601.141135 12615893

[B88] Parker R. Song H. (2004). The enzymes and control of eukaryotic mRNA turnover. *Nat. Struct. Mole. Biol.* 11 121–127. 10.1038/nsmb724 14749774

[B89] Pecot C. V. Calin G. A. Coleman R. L. Lopez-Berestein G. Sood A. K. (2011). RNA interference in the clinic: challenges and future directions. *Nat. Rev. Cancer* 11 59–67. 10.1038/nrc2966 21160526 PMC3199132

[B90] Petrovsky N. Aguilar J. C. (2004). Vaccine adjuvants: current state and future trends. *Immunol. Cell Biol.* 82 488–496. 10.1111/j.0818-9641.2004.01272.x 15479434

[B91] Prud’homme G. J. (2004). Altering immune tolerance therapeutically: the power of negative thinking. *J. Leukoc. Biol.* 75 586–599. 10.1189/jlb.0803394 14657212

[B92] Prud’homme G. J. (2005). DNA vaccination against tumors. *J. Gene Med.* 7 3–17. 10.1002/jgm.669 15538731

[B93] Qiu P. Ziegelhoffer P. Sun J. Yang N. S. (1996). Gene gun delivery of mRNA in situ results in efficient transgene expression and genetic immunization. *Gene Ther.* 3 262–268. 8646558

[B94] Rappuoli R. Mandl C. W. Black S. De Gregorio E. (2011). Vaccines for the twenty-first century society. *Nat. Rev. Immunol.* 11 865–872. 10.1038/nri3085 22051890 PMC7098427

[B95] Rosenblum D. Joshi N. Tao W. Karp J. M. Peer D. (2018). Progress and challenges towards targeted delivery of cancer therapeutics. *Nat. Commun.* 9:1410. 10.1038/s41467-018-03705-y 29650952 PMC5897557

[B96] Sadler A. J. Williams B. R. (2008). Interferon-inducible antiviral effectors. *Nat. Rev. Immunol.* 8 559–568. 10.1038/nri2314 18575461 PMC2522268

[B97] Sanjabi S. Oh S. A. Li M. O. (2017). Regulation of the Immune Response by TGF-β: From Conception to Autoimmunity and Infection. *Cold Spr. Harb. Perspect Biol.* 9: a022236. 10.1101/cshperspect.a022236 28108486 PMC5453394

[B98] Sato Y. Hashiba K. Sasaki K. Maeki M. Tokeshi M. Harashima H. (2019). Understanding structure-activity relationships of pH-sensitive cationic lipids facilitates the rational identification of promising lipid nanoparticles for delivering siRNAs in vivo. *J. Control Release* 295 140–152. 10.1016/j.jconrel.2019.01.001 30610950

[B99] Sautter J. Olesen O. F. Bray J. Draghia-Akli R. (2011). European Union vaccine research–an overview. *Vaccine* 29 6723–6727. 10.1016/j.vaccine.2010.12.060 21195799

[B100] Scheel B. Braedel S. Probst J. Carralot J. P. Wagner H. Schild H. (2004). Immunostimulating capacities of stabilized RNA molecules. *Eur. J. Immunol.* 34 537–547. 10.1002/eji.200324198 14768059

[B101] Schlake T. Thess A. Thran M. Jordan I. (2019). mRNA as novel technology for passive immunotherapy. *Cell Mol. Life Sci.* 76 301–328. 10.1007/s00018-018-2935-4 30334070 PMC6339677

[B102] Schnee M. Vogel A. B. Voss D. Petsch B. Baumhof P. Kramps T. (2016). An mRNA Vaccine Encoding Rabies Virus Glycoprotein Induces Protection against Lethal Infection in Mice and Correlates of Protection in Adult and Newborn Pigs. *PLoS Negl. Trop Dis.* 10:e0004746. 10.1371/journal.pntd.0004746 27336830 PMC4918980

[B103] Shen J. Zhang W. Qi R. Mao Z. W. Shen H. (2018). Engineering functional inorganic-organic hybrid systems: advances in siRNA therapeutics. *Chem. Soc. Rev.* 47 1969–1995. 10.1039/c7cs00479f 29417968 PMC5861001

[B104] Shim M. S. Kwon Y. J. (2012). Stimuli-responsive polymers and nanomaterials for gene delivery and imaging applications. *Adv. Drug. Deliv. Rev.* 64 1046–1059. 10.1016/j.addr.2012.01.018 22329941

[B105] Smaill F. Xing Z. (2014). Human type 5 adenovirus-based tuberculosis vaccine: is the respiratory route of delivery the future? *Exp. Rev. Vacc.* 13 927–930. 10.1586/14760584.2014.929947 24935214

[B106] Staff C. Mozaffari F. Haller B. K. Wahren B. Liljefors M. (2011). A Phase I safety study of plasmid DNA immunization targeting carcinoembryonic antigen in colorectal cancer patients. *Vaccine* 29 6817–6822. 10.1016/j.vaccine.2010.12.063 21195077

[B107] Steitz J. Britten C. M. Wölfel T. Tüting T. (2006). Effective induction of anti-melanoma immunity following genetic vaccination with synthetic mRNA coding for the fusion protein EGFP.TRP2. *Cancer Immunol. Immunother.* 55 246–253. 10.1007/s00262-005-0042-5 16133114 PMC11030217

[B108] Stoitzner P. Tripp C. H. Eberhart A. Price K. M. Jung J. Y. Bursch L. (2006). Langerhans cells cross-present antigen derived from skin. *Proc. Natl. Acad. Sci. U. S. A.* 103 7783–7788. 10.1073/pnas.0509307103 16672373 PMC1472522

[B109] Sum C. H. Nafissi N. Slavcev R. A. Wettig S. (2015). Physical Characterization of Gemini Surfactant-Based Synthetic Vectors for the Delivery of Linear Covalently Closed (LCC) DNA Ministrings. *PLoS One* 10:e0142875. 10.1371/journal.pone.0142875 26561857 PMC4642985

[B110] Tan L. Sun X. (2018). Recent advances in mRNA vaccine delivery. *Nano. Res.* 11 5338–5354. 10.1007/s12274-018-2091-z

[B111] Tanner P. Baumann P. Enea R. Onaca O. Palivan C. Meier W. (2011). Polymeric vesicles: from drug carriers to nanoreactors and artificial organelles. *Acc. Chem. Res.* 44 1039–1049. 10.1021/ar200036k 21608994

[B112] Thess A. Grund S. Mui B. L. Hope M. J. Baumhof P. Fotin-Mleczek M. (2015). Sequence-engineered mRNA Without Chemical Nucleoside Modifications Enables an Effective Protein Therapy in Large Animals. *Mol. Ther.* 23 1456–1464. 10.1038/mt.2015.103 26050989 PMC4817881

[B113] Tiriveedhi V. Fleming T. P. Goedegebuure P. S. Naughton M. Ma C. Lockhart C. (2013). Mammaglobin-A cDNA vaccination of breast cancer patients induces antigen-specific cytotoxic CD4+ICOShi T cells. *Breast Cancer Res. Treat.* 138 109–118. 10.1007/s10549-012-2110-9 22678162 PMC3656506

[B114] Torgerson T. R. Ochs H. D. (2002). Immune dysregulation, polyendocrinopathy, enteropathy, X-linked syndrome: a model of immune dysregulation. *Curr. Opin. Allergy Clin. Immunol.* 2 481–487. 10.1097/00130832-200212000-00002 14752330

[B115] Trimble C. L. Peng S. Kos F. Gravitt P. Viscidi R. Sugar E. (2009). A phase I trial of a human papillomavirus DNA vaccine for HPV16+ cervical intraepithelial neoplasia 2/3. *Clin. Cancer Res.* 15 361–367. 10.1158/1078-0432.Ccr-08-1725 19118066 PMC2865676

[B116] Tseng Y. C. Mozumdar S. Huang L. (2009). Lipid-based systemic delivery of siRNA. *Adv. Drug Deliv. Rev.* 61 721–731. 10.1016/j.addr.2009.03.003 19328215 PMC3172140

[B117] Ulmer J. B. Donnelly J. J. Parker S. E. Rhodes G. H. Felgner P. L. Dwarki V. J. (1993). Heterologous protection against influenza by injection of DNA encoding a viral protein. *Science* 259 1745–1749. 10.1126/science.8456302 8456302

[B118] US National Library Of Medicine ClinicalTrials.gov (2020). *US National Library Of Medicine ClinicalTrials.gov.* Available online at: https://clinicaltrials.gov/ [accessed on Jun 13, 2020].

[B119] Van Craenenbroeck A. H. Smits E. L. Anguille S. Van de Velde A. Stein B. Braeckman T. (2015). Induction of cytomegalovirus-specific T cell responses in healthy volunteers and allogeneic stem cell recipients using vaccination with messenger RNA-transfected dendritic cells. *Transplantation* 99 120–127. 10.1097/tp.0000000000000272 25050468 PMC4281162

[B120] Van Lint S. Renmans D. Broos K. Dewitte H. Lentacker I. Heirman C. (2015). The ReNAissanCe of mRNA-based cancer therapy. *Exp. Rev. Vacc.* 14 235–251. 10.1586/14760584.2015.957685 25263094

[B121] Vasan S. Hurley A. Schlesinger S. J. Hannaman D. Gardiner D. F. Dugin D. P. (2011). In vivo electroporation enhances the immunogenicity of an HIV-1 DNA vaccine candidate in healthy volunteers. *PLoS One* 6:e19252. 10.1371/journal.pone.0019252 21603651 PMC3095594

[B122] Wadhwa A. Aljabbari A. Lokras A. Foged C. Thakur A. (2020). Opportunities and Challenges in the Delivery of mRNA-based Vaccines. *Pharmaceutics* 12:102. 10.3390/pharmaceutics12020102 32013049 PMC7076378

[B123] Wang S. Kennedy J. S. West K. Montefiori D. C. Coley S. Lawrence J. (2008). Cross-subtype antibody and cellular immune responses induced by a polyvalent DNA prime–protein boost HIV-1 vaccine in healthy human volunteers. *Vaccine* 26 3947–3957. 10.1016/j.vaccine.2007.12.060 18724414 PMC3743087

[B124] Wickens M. (1990). How the messenger got its tail: addition of poly(A) in the nucleus. *Trends Biochem. Sci.* 15 277–281. 10.1016/0968-0004(90)90054-f 1974368

[B125] Wilgenhof S. Corthals J. Heirman C. van Baren N. Lucas S. Kvistborg P. (2016). Phase II Study of Autologous Monocyte-Derived mRNA Electroporated Dendritic Cells (TriMixDC-MEL) Plus Ipilimumab in Patients With Pretreated Advanced Melanoma. *J. Clin. Oncol.* 34 1330–1338. 26926680 10.1200/JCO.2015.63.4121

[B126] Wolff J. A. Malone R. W. Williams P. Chong W. Acsadi G. Jani A. (1990). Direct gene transfer into mouse muscle in vivo. *Science* 247 1465–1468. 10.1126/science.1690918 1690918

[B127] Wong S. Lam P. Nafissi N. Denniss S. Slavcev R. (2016). Production of Double-stranded DNA Ministrings. *J. Vis. Exp.* 108:53177. 10.3791/53177 26967586 PMC4828204

[B128] Würtele H. Little K. C. Chartrand P. (2003). Illegitimate DNA integration in mammalian cells. *Gene Ther.* 10 1791–1799. 10.1038/sj.gt.3302074 12960968

[B129] Xiong Q. Lee G. Y. Ding J. Li W. Shi J. (2018). Biomedical applications of mRNA nanomedicine. *Nano. Res.* 11 5281–5309. 10.1007/s12274-018-2146-1 31007865 PMC6472920

[B130] Yamashita A. Chang T.-C. Yamashita Y. Zhu W. Zhong Z. Chen C.-Y. A. (2005). Concerted action of poly(A) nucleases and decapping enzyme in mammalian mRNA turnover. *Nat. Struct. Mole. Biol.* 12 1054–1063. 10.1038/nsmb1016 16284618

[B131] Yang B. Jeang J. Yang A. Wu T. C. Hung C. F. (2014). DNA vaccine for cancer immunotherapy. *Hum. Vaccin Immunother.* 10 3153–3164. 10.4161/21645515.2014.980686 25625927 PMC4514137

[B132] Yang Y. Huang C. T. Huang X. Pardoll D. M. (2004). Persistent Toll-like receptor signals are required for reversal of regulatory T cell-mediated CD8 tolerance. *Nat. Immunol.* 5 508–515. 10.1038/ni1059 15064759

[B133] Yankauckas M. A. Morrow J. E. Parker S. E. Abai A. Rhodes G. H. Dwarki V. J. (1993). Long-term anti-nucleoprotein cellular and humoral immunity is induced by intramuscular injection of plasmid DNA containing NP gene. *DNA Cell Biol.* 12 771–776. 10.1089/dna.1993.12.771 7692877

[B134] Yoo J. W. Irvine D. J. Discher D. E. Mitragotri S. (2011). Bio-inspired, bioengineered and biomimetic drug delivery carriers. *Nat. Rev. Drug Discov.* 10 521–535. 10.1038/nrd3499 21720407

[B135] Younger D. S. Younger A. P. Guttmacher S. (2016). Childhood Vaccination: Implications for Global and Domestic Public Health. *Neurol. Clin.* 34 1035–1047. 10.1016/j.ncl.2016.05.004 27719987

[B136] Yuan J. Ku G. Y. Gallardo H. F. Orlandi F. Manukian G. Rasalan T. S. (2009). Safety and immunogenicity of a human and mouse gp100 DNA vaccine in a phase I trial of patients with melanoma. *Cancer Immun.* 9:5. PMC2888533 19496531

[B137] Zafrir Y. Agmon-Levin N. Paz Z. Shilton T. Shoenfeld Y. (2012). Autoimmunity following hepatitis B vaccine as part of the spectrum of ‘Autoimmune (Auto-inflammatory) Syndrome induced by Adjuvants’ (ASIA): analysis of 93 cases. *Lupus* 21 146–152. 10.1177/0961203311429318 22235045

