# Supplementary material for: A Guide to Nucleic Acid Vaccines in the Prevention and Treatment of Infectious Diseases and Cancers: From Basic Principles to Current Applications
Source: Front Cell Dev Biol. 2021 May 25;9:633776. doi: 10.3389/fcell.2021.633776 (PMC8185206; doi:10.3389/fcell.2021.633776)
Supplement: Supplementary file 1 [file Table_1.docx]

**Table 1: Clinical trials of nucleic acid vaccines against infectious diseases**

| Types(DNA vaccine) | Disease | Trial numbers | Phase | Status | Administration | Sponsors |
| --- | --- | --- | --- | --- | --- | --- |
| pGA2/JS2 | HIV | [NCT00043511](http://clinicaltrials.gov/show/NCT00043511) | I | Completed | Intramuscular injection | National Institute of Allergy and Infectious Diseases (NIAID) |
| VCL-IPT1andVCL-IPM1 | Pandemic influenza | [NCT00709800](http://clinicaltrials.gov/show/NCT00709800) | I | Completed | Intramuscular injection | Vical |
| VRC-EBODNA023-00-VP and VRC-MARDNA025-00-VP | Ebola and Marburg virus | [NCT00605514](http://clinicaltrials.gov/show/NCT00605514) | I | Completed | Intramuscular injection | NIAID |
| INO-4212 and With or Without INO-9012 | Ebola | [NCT02464670](http://clinicaltrials.gov/show/NCT02464670) | I | Completed | Intradermal or Intramuscular injection followed by electroporation | Inovio Pharmaceuticals |
| GLS-5700 | Dengue virus seropositive adults | [NCT02887482](http://clinicaltrials.gov/show/NCT02887482) | I | Completed | EP-enhanced intradermal (ID) injection | GeneOne Life Science, Inc. |
| pCMVS2-S | Chronic B Hepatitis | [NCT00536627](http://clinicaltrials.gov/show/NCT00536627) | I ,II | Completed | Intramuscular injection | French National Agency for Research on AIDS and Viral Hepatitis |
| HB-110 | Hepatitis B | NCT00513968 | I | Completed | Intramuscular injection | Genexine, Inc |

**Table 1(cont.): Clinical trials of nucleic acid vaccines against infectious diseases**

| Types(DNA vaccine) | Disease | Trial numbers | Phase | Status | Administration | Sponsors |
| --- | --- | --- | --- | --- | --- | --- |
| HBV-DNA plasmid pdpSC18 vaccine | Hepatitis B | NCT00277576 | I | Completed | Intramuscular injection | PowderMed/Pfizer |
| VCL-HB01 | Genital Herpes Simplex Type 2 | NCT02030301 | I,II | Completed | Intramuscular injection | Vical |
| pPJV7630 | HSV-2 | NCT00274300 | I | Completed | particle-mediated epidermal delivery (PMED) | PowderMed |
| EP HIV-1090 | HIV-1(AIDS) | NCT00054860 | I | Completed | Intramuscularly | NIAID |
| VRC-HIVDNA009-00-VP | HIV-2(AIDS) | NCT00125099 | I, II | Completed | Administered to individuals who started antiretroviral therapy during acute HIV-1 infection | NIAID |
| VGX-6150-01 | Hepatitis C, Chronic | NCT02027116 | I | Completed | Intramuscular injection with Electroporation | GeneOne Life Science, Inc |
| VRC-FLUDNA061-00-VP | Influenza: H1N1, H3N2 | NCT01498718 | Ib | Completed | Intramuscular injection | NIAID |
| VRC-SRSDNA015-00-VP | SARS | NCT00099463 | I | Completed | Intramuscular injection | NIAID |
|  |  |  |  |  |  |  |

**Table 1(cont.): Clinical trials of nucleic acid vaccines against infectious diseases**

| Types(DNA vaccine) | Disease | Trial numbers | Phase | Status | Administration | Sponsors |
| --- | --- | --- | --- | --- | --- | --- |
| Prime Boost Regimen of DNA- and adenovirus-vectored malaria vaccine encoding Plasmodium falciparum circumsporozoite protein | Malaria | NCT00870987 | I, II | Completed | Subcutaneous injection | U.S. Army Medical Research and Development Command |
| Ad5Ag85A recombinant replication-deficient adenoviral vector | Tuberculosis | NCT00800670 | I | Terminated | Intramuscular injection | McMaster University |
| DNA plasmid vaccine | COVID-19 | NCT04336410 | I | Active, not recruiting | Electroporation device | Inovio Pharmaceuticals |
| VRC-WNVDNA020-00-VP | West Nile virus infection | NCT00300417 | I | Completed | Intramuscularly using the Biojector 2000 Needle-Free Injection Management System | NIAID |

**Table 1(cont.): Clinical trials of nucleic acid vaccines against infectious diseases**

| Types(RNA vaccine) | Disease | Trial numbers | | Phase | Status | Administration | Sponsors |
| --- | --- | --- | --- | --- | --- | --- | --- |
| DC EP with autologous viral  Ag and CD40L mRNAs | HIV-1 | NCT00672191 | II | | Completed | Intradermal injection | Argos Therapeutics |
|  |  | NCT01069809 | II | | Completed |  |  |
|  |  | NCT02042248 | I | | Completed |  |  |
| DC loaded with viral Ag mRNA with TriMix |  | NCT02888756 | II | | Terminated | Intranodal injection | Erasmus Medical Center |
| Viral Ag mRNA with TriMix |  | NCT02413645 | I | | Completed | Not available | Fundació Clínic per la Recerca  Biomèdica |
| DC loaded with viral Ag mRNA |  | NCT00833781 | II | | Completed | Intradermal injection | Massachusetts General Hospital |
| DC EP with autologous viral  Ag and CD40L mRNAs |  | NCT00381212 | I, II | | Completed | Intradermal injection | McGill University Health Centre |
| RNActive viral Ag mRNA | Rabies virus | NCT02241135 | I | | Completed | Intradermal injection, Intramuscular injection | CureVac AG |
| CV7202 |  | NCT03713086 | I | | Active, not recruiting | Intramuscular injection | CureVac |
|  |  |  |  | |  |  |  |

**Table 1(cont.): Clinical trials of nucleic acid vaccines against infectious diseases**

| Types(RNA vaccine) | Disease | Trial numbers | Phase | Status | Administration | Sponsors |
| --- | --- | --- | --- | --- | --- | --- |
| mRNA‐1893 | Zika virus | NCT04064905 | I | Active, not recruiting | Not specified | Moderna/Biomedical  Advanced Research and Development Authority |
| Nucleoside-modified viral Ag  mRNA |  | NCT03014089 | I, II | Completed | Intramuscular injection | Moderna  Therapeutics |
| mRNA‐1653 | Human metapneumovirus Human Parainfluenza virus | NCT03392389 | I | Completed | Not specified | ModernaTX, Inc |
|  |  | NCT03829384 | I | Active, not recruiting | Parenteral |  |
| mRNA‐1944 | Chikungunya virus | NCT04144348 | I | Recruiting | Parenteral | Moderna |
| mRNA-1273 | COVID-19 | NCT04283461 | I | Active, not recruiting | Intramuscular | Moderna/NIAID |
|  |  |  |  |  |  |  |
