# Supplementary material for: A Guide to Nucleic Acid Vaccines in the Prevention and Treatment of Infectious Diseases and Cancers: From Basic Principles to Current Applications
Source: Front Cell Dev Biol. 2021 May 25;9:633776. doi: 10.3389/fcell.2021.633776 (PMC8185206; doi:10.3389/fcell.2021.633776)
Supplement: Supplementary file 2 [file Table_2.docx]

**Table 2: Clinical trials of nucleic acid vaccines against cancer**

| Types(DNA vaccine) | Disease | Trial numbers | Phase | Status | Administration | Sponsors |
| --- | --- | --- | --- | --- | --- | --- |
| INO-3112 | Cervical Cancer | [NCT02172911](http://clinicaltrials.gov/show/NCT02172911) | II | Completed | Intramuscular injection, Electroporation | Inovio Pharmaceuticals |
| Fourth dose of Human Papillomavirus (HPV) DNA plasmid (VGX-3100) + EP in adult females previously vaccinated with three doses of VGX-3100 |  | NCT01188850 | I | Completed | Intramuscular injection, Electroporation using CELLECTRA device |  |
| CD105/Yb 1/SOX2/CDH3/MDM2-polyepitope Plasmid DNA Vaccine | Patients with HER2-Negative Stage III-IV Breast Cancer | [NCT00831467](http://clinicaltrials.gov/show/NCT00831467) | I, II | Completed | Intradermal injection | University of Washington |
| Neoantigen DNA Vaccine | Pancreatic Cancer | [NCT03122106](http://clinicaltrials.gov/show/NCT03122106) | I | Active, not recruiting | Intramuscular, injection, and electroporation | Washington University School of Medicine |
| pVAXrcPSAv53l | Prostate Cancer | [NCT00859729](http://clinicaltrials.gov/show/NCT00859729) | I, II | Completed | Intradermal injection and Electroporation | Uppsala University |
| pTVG-HP | Non-metastatic Prostate Cancer | [NCT01341652](http://clinicaltrials.gov/show/NCT01341652) | II | Completed | Intradermal injection | University of Wisconsin, Madison |

**Table 2(cont.): Clinical trials of nucleic acid vaccines against cancer**

| Types(RNA vaccine) | Disease | Trial numbers | Phase | Status | Administration | Sponsors |
| --- | --- | --- | --- | --- | --- | --- |
| DC EP with TAA mRNA | Glioblastoma | NCT02649582 | I, II | Recruiting | Intradermal injection or Not Available | Antwerp University Hospital |
|  | Mesothelioma | NCT02649829 | I, II | Recruiting |  |  |
|  | Multiple solid tumors | NCT01291420 | I, II | Unknown |  |  |
|  | AML, CML, multiple myeloma | NCT00965224 | II | Unknown |  |  |
|  | AML | NCT01686334 | II | Recruiting |  |  |
|  |  | NCT00834002 | I | Completed |  |  |
| DC loaded with TAA mRNA | AML | NCT00510133 | II | Completed | Not Available | Asterias Biotherapeutics |
| DC loaded with TAA and CMV Ag mRNA |  | NCT01734304 | I, II | Completed | Intradermal injection | Ludwig-Maximilian- University of Munich |
| DC loaded with AML lysate and mRNA |  | NCT00514189 | I | Terminated | Not Available | MD Anderson Cancer  Center |
|  |  |  |  |  |  |  |

**Table 2(cont.): Clinical trials of nucleic acid vaccines against cancer**

| Types(RNA vaccine) | Disease | Trial numbers | Phase | Status | Administration | Sponsors |
| --- | --- | --- | --- | --- | --- | --- |
| DC EP with autologous tumor  mRNA with or without CD40L mRNA | Renal cell carcinoma | NCT01482949 | II | Terminated | Intradermal injection | Argos Therapeutics |
|  |  | NCT00678119 | II | Completed |  |  |
|  |  | NCT00272649 | I, II | Completed |  |  |
|  |  | NCT01582672 | III | Terminated |  |  |
|  |  | NCT00087984 | I, II | Completed |  |  |
| Liposome-formulated TAA and neo-Ag mRNA | Breast cancer | NCT02316457 | I | Active, not recruiting | Intravenous injection | BioNTech RNA  Pharmaceuticals GmbH |
| Liposome-complexed TAA mRNA | Melanoma  Breast cancer  Melanoma | NCT02410733 | I | Active, not recruiting | Intravenous injection |  |
| Naked TAA or neo-Ag mRNA |  | NCT01684241 | I | Completed | Intranodal injection |  |
|  |  | NCT02035956 | I | Completed |  |  |
| DC EP with TAA and TriMix mRNA |  | NCT01066390 | I | Completed | Intradermal injection and Intravenous injection | Universitair Ziekenhuis Brusse |
|  |  | NCT01302496 | II | Completed |  |  |
|  |  | NCT01676779 | II | Completed |  |  |

**Table 2(cont.): Clinical trials of nucleic acid vaccines against cancer**

| Types(RNA vaccine) | Disease | Trial numbers | Phase | Status | Administration | Sponsors |
| --- | --- | --- | --- | --- | --- | --- |
| Autologous tumor mRNA with GM-CSF protein | Melanoma  Breast cancer  Melanoma | NCT00204516 | I, II | Completed | Intradermal injection and Subcutaneous injection | University Hospital  Tübingen |
| Protamine-complexed TAA mRNA with GM-CSF protein |  | NCT00204607 | I, II | Completed | Intradermal injection and Subcutaneous injection |  |
|  |  | NCT00929019 | I, II | Terminated |  | Radboud University |
|  |  | NCT00243529 | I, II | Completed |  |  |
|  |  | NCT00940004 | I, II | Completed |  |  |
|  |  | NCT01530698 | I, II | Completed |  |  |
|  |  | NCT02285413 | II | Completed |  |  |
|  | Colorectal cancer | NCT00228189 | I, II | Completed |  |  |
| RNActive TAA mRNA | Non-small-cell lung cancer | NCT00923312 | I, II | Completed | Intradermal injection | CureVac AG |
|  |  | NCT01915524 | I | Terminated |  |  |

**Table 2(cont.): Clinical trials of nucleic acid vaccines against cancer**

| Types(RNA vaccine) | Disease | Trial numbers | Phase | Status | Administration | | | Sponsors |
| --- | --- | --- | --- | --- | --- | --- | --- | --- |
| RNActive TAA mRNA | Prostate Cancer | NCT02140138 | II | Terminated | Intradermal injection | CureVac AG | | |
|  |  | NCT00831467 | I, II | Completed |  |  |  |  |
|  |  | NCT01817738 | I, II | Terminated |  |  |  |  |
| RNActive* TAA mRNA |  | NCT00906243 | I, II | Terminated | Intradermal injection | | | University of Florida |
| DC loaded with TAA mRNA |  | NCT01446731 | II | Completed | Intradermal injection | | | Herlev Hospital |
|  | Breast cancer, melanoma | NCT00978913 | I | Completed | Intradermal injection | | | Herlev Hospital |
| DC loaded with CMV Ag mRNA with GM-CSF protein | Glioblastoma, malignant glioma | NCT02465268 | II | Recruiting | Intradermal injection | | | University of Florida |
| DC loaded with CMV Ag mRNA |  | NCT00626483 | I | Completed | Intradermal injection or inguinal injection. | | Duke University | |
|  |  | NCT00639639 | I | Active, not recruiting |  |  |  |  |
|  |  | NCT02529072 | I | Completed |  |  |  |  |
|  |  | NCT02366728 | II | Active, not recruiting |  |  |  |  |

**Table 2(cont.): Clinical trials of nucleic acid vaccines against cancer**

| Types(RNA vaccine) | Disease | Trial numbers | Phase | Status | Administration | Sponsors |
| --- | --- | --- | --- | --- | --- | --- |
| DC loaded with TAA mRNA | Brain metastases | NCT02808364 | I, II | Active, not recruiting | Not Available | Guangdong 999 Brain  Hospital |
|  |  | NCT02709616 | I, II |  |  |  |
|  |  | NCT02808416 | I, II |  |  |  |
| DC, matured, loaded with TAA mRNA | Melanoma | NCT01216436 | I | Terminated | Intranodal injection | Duke University |
| DC (Langerhans) EP with TAA mRNA |  | NCT01456104 | I | Active, not recruiting | Intradermal injection | Memorial Sloan Kettering Cancer Center |
| Multiple myeloma |  | NCT01995708 | I | Active, not recruiting | Intradermal injection |  |
| DC, matured, loaded with TAA mRNA |  | NCT01456065 | I | Unknown | Not Available | Life Research  Technologies GmbH |
| DC loaded with TAA mRNA | AML, myelodysplastic syndromes | NCT03083054 | I, II | Active, not recruiting | Not Available | University of Campinas, Brazil |
| W_ova1 vaccine | Ovarian cancer | NCT04163094 | I | Recruiting | Intravenous | University Medical Center Groningen/BioNTech |
|  |  |  |  |  |  |  |

**Table 2(cont.): Clinical trials of nucleic acid vaccines against cancer**

| Types(RNA vaccine) | Disease | Trial numbers | Phase | Status | Administration | Sponsors |
| --- | --- | --- | --- | --- | --- | --- |
| DC loaded with autologous tumor or TAA mRNA | Melanoma | NCT00961844 | I, II | Terminated | Intradermal injection or Not Available | Oslo University Hospital |
|  |  | NCT01278940 | I, II | Completed |  |  |
|  | Prostate Cancer | NCT01197625 | I, II | Active, not recruiting |  |  |
|  |  | NCT01278914 | I, II | Completed |  |  |
|  | Glioblastoma | NCT00846456 | I, II | Completed |  |  |
|  | Ovarian cancer | NCT01334047 | I, II | Terminated |  |  |
| Immunization with  DCs loaded with  MiHA mRNA | Hematological malignancies | NCT02528682 | I, II | Recruiting | Intravenous  Injection | The Netherlands Organization for Health Research and Development Dutch Cancer Society |
| mRNA‐4157 | Solid tumors | NCT03313778 | I | Recruiting | Not specified | Merck Sharp&Dohme |
|  | Cutaneous melanoma | NCT03897881 | II | Recruiting | Not specified |  |

**Table 2(cont.): Clinical trials of nucleic acid vaccines against cancer**

| Types(RNA vaccine) | Disease | Trial numbers | Phase | Status | Administration | Sponsors |
| --- | --- | --- | --- | --- | --- | --- |
| Personalized mRNA | Advanced esophageal squamous carcinoma, gastric adenocarcinoma, pancreatic adenocarcinoma, colorectal adenocarcinoma | NCT03468244 | Enrolling | Recruiting | Subcutaneous injection | Changhai Hospital/Stemirna Therapeutics |
|  | Esophageal cancer, Non‐small cell lung cancer, | NCT03908671 | Not Applicable | Not yet recruiting |  | Stemirna Therapeutics |
| mRNA[BI1361849(formerly CV9202)] | Metastatic non‐small cell lung cancer | NCT03164772 | I, II | Recruiting | Not specified | Ludwig Institute for Cancer Research |
| mRNA‐5671/V941 | Neoplasms, carcinoma, non-small-cell lung, pancreatic neoplasms,  colorectal neoplasms | NCT03948763 | I | Recruiting | Intramuscular injection | Merck Sharp&Dohme |
|  |  |  |  |  |  |  |

**Table 2(cont.): Clinical trials of nucleic acid vaccines against cancer**

| Types(RNA vaccine) | Disease | Trial numbers | Phase | Status | Administration | Sponsors |
| --- | --- | --- | --- | --- | --- | --- |
| mRNA‐2416 | Relapsed/Refractory Solid Tumor Malignancies or Lymphoma,  Ovarian Cancer | NCT03323398 | I, II | Recruiting | Intratumoral | ModernaTX, Inc. |
| mRNA‐2752 | Dose Escalation: Relapsed/Refractory Solid Tumor Malignancies or Lymphoma  Dose  Expansion: Triple Negative Breast Cancer, Head and Neck Squamous Cell Carcinoma, Non-Hodgkin Lymphoma, and Urothelial Cancer | NCT03739931 | I | Recruiting | Intratumoral | Moderna/  AstraZeneca |

**Table 2(cont.): Clinical trials of nucleic acid vaccines against cancer**

| Types(RNA vaccine) | Disease | Trial numbers | Phase | Status | Administration | Sponsors |
| --- | --- | --- | --- | --- | --- | --- |
| DC EP with autologous tumor  mRNA with or without CD40L mRNA | Pancreatic cancer | NCT00664482 | Not Available | Completed | Not Available | Argos Therapeutics |
| DC, matured, loaded with autologous tumor RNA | Melanoma,Breast cancer,Melanoma | NCT01983748 | III | Recruiting | Intravenous injection | University Hospital  Erlangen |
| DC loaded with autologous tumor mRNA | Glioblastoma | NCT00890032 | I | Completed | Intradermal injection | Duke University |
| Human CMV pp65‐LAMP mRNA | Glioblastoma | NCT03927222 | II | Recruiting | Intradermal | Gary Archer Ph.D./Duke University |
|  |  |  |  |  |  |  |
